# Time to get moving: assisted gene flow of forest trees

**DOI:** 10.1111/eva.12293

**Published:** 2015-08-24

**Authors:** Sally N. Aitken, Jordan B. Bemmels

**Affiliations:** ^1^ Department of Forest and Conservation SciencesUniversity of British ColumbiaVancouverBCCanada; ^2^ Department of Ecology and Evolutionary BiologyUniversity of MichiganAnn ArborMIUSA

**Keywords:** assisted migration, climate change, ecological genetics, forest policy, genetic clines, local adaptation, provenance, temperate species

## Abstract

Geographic variation in trees has been investigated since the mid‐18th century. Similar patterns of clinal variation have been observed along latitudinal and elevational gradients in common garden experiments for many temperate and boreal species. These studies convinced forest managers that a ‘local is best’ seed source policy was usually safest for reforestation. In recent decades, experimental design, phenotyping methods, climatic data and statistical analyses have improved greatly and refined but not radically changed knowledge of clines. The maintenance of local adaptation despite high gene flow suggests selection for local adaptation to climate is strong. Concerns over maladaptation resulting from climate change have motivated many new genecological and population genomics studies; however, few jurisdictions have implemented assisted gene flow (AGF), the translocation of pre‐adapted individuals to facilitate adaptation of planted forests to climate change. Here, we provide evidence that temperate tree species show clines along climatic gradients sufficiently similar for average patterns or climate models to guide AGF in the absence of species‐specific knowledge. Composite provenancing of multiple seed sources can be used to increase diversity and buffer against future climate uncertainty. New knowledge will continue to refine and improve AGF as climates warm further.

## Introduction

Long‐lived, largely undomesticated and slow to reach reproductive maturity, forest trees have never been ideal genetic subjects. As a result, genetic knowledge of trees has lagged behind that of agricultural crops and model organisms. However, one area in which knowledge of genetic variation within forest trees has led many other life forms for over two centuries is genecology, the study of relationships between natural populations and their native environments. The economic importance of trees for wood and fibre combined with their ecological importance as foundation species motivated scientists to characterize geographic variation as a prerequisite for deciding what to plant where. The synchronization of growth and dormancy timing of trees with their local climate was recognized early on to be a prerequisite for successful reforestation and led to a general practice of using locally collected seed for reforestation. A rapidly warming global climate is now disrupting adaptation of local populations, and the long, historically appropriate conservative tradition of ‘local is best’ has been slow to evolve to address this new challenge.

**Figure 1:**
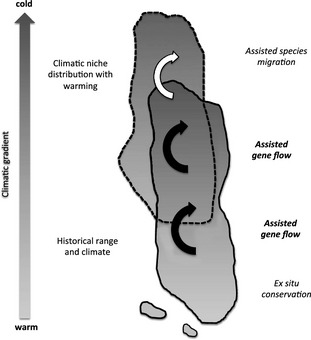
Schematic diagram of management options for reforestation and restoration in a changing climate. While this illustrates the northward movement of individuals, assisted gene flow may also occur along elevational or longitudinal climatic gradients.

The future health and productivity of tree populations will depend on the match between genotypes and new environments. Contemporary scientists are addressing these questions using traditional common garden approaches, as well as new phenotyping and genomic methods, to assess capacity for adaptation to new climates and inform ‘assisted gene flow’ (AGF), the managed translocation of individuals within the current species range to facilitate rapid adaptation to climate change (Aitken and Whitlock 2013) (Fig. 1). Here, we argue that we already have sufficient knowledge from a rich history of research to initiate AGF of temperate and boreal forest trees. We do not extend our analysis or recommendations to tropical species as there are fewer data available on local adaptation and the ecological context differs greatly.

Göte Turesson (1923) coined the term genecology and defined it as ‘race ecology’, the study of ‘hereditary variation in relation to habitat’, emphasizing the ecological rather than genetic content of the definition. The current use of genecology is largely synonymous with the more widely used term ecological genetics, although Langlet (1971) argued Turesson intended for the former to emphasize ecology, while the latter emphasizes genetics. Genecology is used frequently by many forest geneticists; most other botanists use ecological genetics, perhaps because genecology autocorrects in most software to a branch of gender‐specific medicine. The French term ‘provenance’ has long been used in forestry to describe the place of origin of genetic material and is often used interchangeably with population, the interbreeding individuals of a species at a location. In this study, we will use genecology and provenance to honour a long and rich tradition in forest biology.

Here, we review the history of genecological research in forest trees, emphasizing the relationships between populations and climate. We then highlight advances in genecology and other relevant fields in recent decades; summarize key findings from population genetics and genomics relevant to local adaptation; present data on the concordance of genetic clines among tree species in western North America; and discuss the implications for managing tree populations in a rapidly warming world. This study builds on recent reviews and syntheses by the senior author (Box 1) and others describing the range of potential responses of forest trees to climate change and the factors controlling them (Aitken et al. 2008); the potential for evolutionary and plastic responses to climate change in trees (Alberto et al. 2013) and plants in general (Franks et al. 2014); and the genetic risks and benefits of AGF (Aitken and Whitlock 2013).

Box 1 **Personal reflections on and lessons from a career in forest genetics – Sally Aitken^1^**

I knew I wanted to be an evolutionary biologist from an early age. When I was 5 years old, my family spent a month camping at the Burgess Shale, while my father worked as part of an international team of geologists to quarry for branch origins in the tree of life in one of the world's most important fossil beds. At the age of 12, my father encouraged me to read Darwin's “*On the Origin of Species*”. This was a transformative experience that drove my career interests in genetics and evolutionary biology. Guessing that genetics might matter in forestry, and wanting to work in an applied field, I started the Forest Resources Management program in the Faculty of Forestry at the University of British Columbia (UBC) in 1979.

**Don't avoid male‐dominated fields**

The almost complete male domination of forestry in the 1970s did not dissuade me, thanks to my parents’ influence. My mother had a degree in agriculture and had worked in various research labs prior to starting a family, and through my father I met many scientists, mostly men but a few women. At sixteen, when looking for a part‐time job that would accommodate ski racing, I observed that only guys seemed to work as ski technicians. On hearing this, my father said he never wanted to hear me say I couldn't do something because I was female. I applied at three ski shops, and was offered three jobs.

**The value of chance meetings**

At UBC, there were no female professors in the Faculty of Forestry while I was an undergraduate. However, my cohort had had a higher proportion of female students than ever before, and the environment was supportive. A summer job in 1981 working on silvicultural research trials near Prince George, BC, landed me by chance next to Jon Dietrichson, a Norwegian forest geneticist, on a field trip bus. This encounter started my career in forest genetics; he hired me the next year as a Trainee in Tree Breeding at the Norwegian Forest Research Institute. I recommend that students and postdocs talk to people they don't know at conference coffee breaks, receptions and field trips, as these encounters might open doors unexpectedly.

**The value of mentors**

In 1984, I started graduate studies with W.J. (Bill) Libby at the University of California, Berkeley. Bill mentored many people, both women and men, in forest genetics. He was a prolific source of new ideas and something of a futurist in predicting technologies, and he taught me to think broadly. I had many other mentors in Berkeley, including Connie Millar, who completed her PhD with Bill Libby a few years ahead of me in conifer population and conservation genetics, and is now a well known expert on trees and climate change. Tom Ledig and Tom Conkle were generous with their advice and with their laboratory facilities at the USDA Forest Service Southwest Research Station. They hosted a weekly forest genetics coffee with W.B. (Bill) Critchfield, who inspired me to study the origins of *Pinus contorta* ssp. *bolanderi* in the Mendocino pygmy forest. An internship at the historic Institute of Forest Genetics with postdoctoral fellows Claire Kinlaw and David Neale in the early days of DNA sequencing helped me decide I did not want to become a molecular biologist, but instead wanted to stick with population, quantitative and ecological genetics. It is more than a little ironic that genomic methods now play a large role in my research.

**The value of applied experience and collaboration**

My first post‐doctoral academic position was at Oregon State University, as first a Research Associate then as Research Assistant Professor with the Pacific Northwest Tree Improvement Research Cooperative, working with Director and Professor W. T. Adams (1990–1996). I gained experience in tree breeding and quantitative genetics, and developed a better understanding of seasonal growth and dormancy cycles through repeatedly phenotyping field common gardens. I also developed more fluency in operational forestry, which later helped me establish collaborations, successfully obtain applied funding, and identify genetic questions that addressed applied problems. Close collaborators included Katy Kavanagh, Barbara Bond, Barbara Lauchenbrach, and Les Fuchigami, all of whom taught me phenotyping techniques for drought and cold hardiness. I also learned how not to do some things: how not to be relegated to the role of a research assistant while an assistant professor; and how not to get bogged down by administration and service at the expense of publishing. My daughter was an infant when I started this position, and balancing work and motherhood was challenging. It gets much easier to publish and parent simultaneously with time. My daughter is now a PhD student herself, and I learn a great deal from her.

**Good fortune and good people**

In academia, we get little say in where a tenure‐track offer might come from, so it felt like winning a lottery when I was offered the job of my dreams, an NSERC Industry Chair at my *alma mater* in 1996. Given my research record at the time, my department took a chance on me. In the 18 years since then I have been very fortunate to have hardworking, intelligent and kind graduate students, postdocs, research associates and research assistants; supportive colleagues; productive and thoughtful collaborators; and good research funding. We started the Centre for Forest Conservation Genetics in 2001, and I owe much to our talented Associate Director Tongli Wang and lab and project manager Pia Smets. Half the professors in my Department are now women, and our undergraduate students in Forest Resources Management, Forest Sciences, and Natural Resources Conservation are close to gender balanced. Judith Myers (featured in this special issue) has been a key mentor for me and many other women scientists at the University of British Columbia. The large‐scale AdapTree genomics project has allowed me to collaborate in an emerging area of science with a group of outstanding evolutionary biologists, and they have taught me a great deal. My partner Jack Woods is very supportive of my career, which has been an enormous help. While subconscious gender bias in science and academia remains common, I increasingly see both male and female colleagues working cooperatively against it. One important lesson I've learned over my career is what really matters for wellbeing and productivity is maintaining a positive and supportive atmosphere in my research group, and choosing collaborators carefully. Finally, at this career stage, great pleasure comes from seeing young scientists I have worked with progress in their careers in the directions they desire.

1This paper has been written for a special issue featuring women in evolutionary biology; and these personal reflections are in response to a request by the editors.

## Early studies of geographic variation in forest trees

Scientists have studied relationships between tree populations and environmental characteristics of their provenances in common garden experiments for over 250 years, long before Clausen et al. (1940, 1948) conducted their much‐cited reciprocal transplant experiments in California with the herbaceous perennial *Achillea millefolium* and other species. Olof Langlet (1971) compiled a detailed history of genecology, and this brief historical summary is largely derived from his work.

The need to grow trees for timber, particularly for shipbuilding, drove pioneers in forest genetics to seek optimal seed sources for planting. H.L. Duhamel du Monceau, the Inspector‐General of the French Navy and a forest botanist, grew Scots pine (*Pinus sylvestris*) from Central Europe, Russia, Scotland and the Baltic region on his estate in the 1740s and 1750s, but the results were never published (Langlet 1971). Around the same time, North American tree species were being evaluated for use in Europe, and von Wagenheim (1787) emphasized the importance of considering provenance climate and soils within the native range when selecting seed sources for either montane or lowland German planting sites. The need for naval timber also motivated Patrick Matthew's (1831) little‐known book *On Naval Timber and Arboriculture*, in the appendix of which he published a theory of natural selection 28 years before Charles Darwin.

Pierre Philippe André de Vilmorin repeated the earlier work of Duhamel in France in the 1820s, establishing a provenance study of Scots pine on his own estate that included seed collections from across Europe and Russia comprising all previously described varieties and closely related species. Vilmorin (1862, in Langlet 1971) was the first to recognize the continuous nature of intraspecific variation among populations across geographic areas and environmental gradients, rather than distinct taxonomic or morphological varieties that might be described if only populations at the ends of these gradients were sampled. This work was highly influential in European forestry.

Charles Darwin was a keen observer of tree biology among other things. In *On the Origin of Species,* he referenced William Hooker's study of Himalayan pines and rhododendrons collected from provenances at different elevations and observed they “possess different constitutional powers of resisting cold” when grown at Kew Gardens in London (Darwin 1859). Darwin also recognized the high lifetime fecundity of long‐lived trees, and the great opportunity for selection among seedlings this presented each generation. He marvelled at the inefficiency of wind‐dispersed pollen in many tree species, but it was more than a century before studies with genetic markers revealed the extent to which this results in high levels of gene flow and within‐population variation, providing variation each generation for local adaptation (Kremer et al. 2012).

Awareness of the importance of geographic variation and adaptation of trees to climate was also accruing from plantation failures. Reports of plantation collapse attributed to ‘foreign’ Scots pine seed in Austria, Denmark, Finland, Lithuania and Norway were reported between 1878 and 1904, and the government of Sweden officially warned against import of non‐Swedish seed sources of Norway spruce and Scots pine in 1882 (Langlet 1971). By the end of the 19th century, the need to consider seed provenance was well established in European forestry. The International Union of Forest Research Organizations was founded in 1892 and has facilitated the exchange of genetic material and the establishment of international collaborations for provenance testing since that time (Krutzsch 1992). Adapting forests to climate change will require continued or renewed international collaboration for exchange of climatically suitable genetic material.

While early observations were from single test sites or from anecdotal observations of plantations, multi‐site provenance trials were developed by the end of the 19th century. Cieslar studied needle characteristics, growth phenology and cold injury, mostly along elevational gradients in the Austrian Alps. He concluded that within species and recognized morphological varieties, there are physiological varieties that have locally adapted to specific site factors over long time periods (Cieslar 1895, 1899; Langlet 1971). In Switzerland, Engler (1908) recognized that the extensive, continuous and gradual variation in Scots pine across Europe lacked any sharp boundaries.

In summary, by the end of the 1800s it was fairly widely recognized in Europe that (i) temperate and boreal tree species vary geographically in a manner corresponding to their provenance climate of origin, (ii) trees need to be planted in common gardens to observe this variation, (iii) most variation is continuous along environmental gradients rather than discrete or ecotypic, (iv) variation is more often physiological (e.g. in growth rate or phenology) than morphological and involves tradeoffs between growth rate and cold tolerance in many cases, and (v) seed for reforestation should in most cases be procured locally to ensure adequate growth and hardiness. More recent studies improved on experimental design, multiple sites or environments, phenotyping methods, and sources of climate data for provenance trials, and produced a more nuanced understanding of the genetic influence of provenance, the environmental effects of experimental environments and their interactions. New population genomic analyses are generating a better understanding of the genetic architecture of climate‐related traits and confirming geographic patterns of local adaptation.

## Climate‐related phenotypic traits

Patterns of variation in phenotypic traits with strong signatures of local adaptation to climate should be used to modify seed transfer practices for reforestation and implement AGF, the translocation of individuals to facilitate adaptation to climate (Aitken and Whitlock 2013). The strength of population divergence and local adaptation for phenotypic traits is typically estimated as *Q*_ST_, the proportion of total genetic variation due to among‐population variation (Spitze 1993; Howe et al. 2004) or as the *Q*_ST_ proxy *V*_pop_, the proportion of phenotypic variation due to among‐population variation (Alberto et al. 2013). Traits with *Q*_ST_ estimates significantly higher than *F*_ST_ for selectively neutral genetic markers are likely under divergent selection in different populations (Whitlock 2008; Whitlock and Guillaume 2009). While *Q*_ST_ and *V*_pop_ describe relative amounts of among‐population variation, they do not identify specific environmental factors driving population differentiation. Environmental factors associated with population differentiation can be evaluated based on the strength of genetic clines in phenotypes in a common garden, assuming that average phenotypes vary due to natural selection and reflect local adaptation to environmental gradients. They can also be assessed as those factors resulting in genotype‐by‐environment interaction in reciprocal transplant experiments, but differences between field sites are often not attributable to a single environmental factor.

### Growth as a proxy for fitness

Foresters have used growth rate as the primary phenotypic trait of interest in provenance trials for practical reasons. Height and diameter can quickly be measured and used to estimate wood volume. Growth has also been widely used as a proxy for juvenile fitness, as lifetime fitness and reproduction estimates are not feasible for organisms that outlive their students. Trees must survive and remain healthy to grow large; trees that are taller compete more effectively for light and are more likely to survive the strong density‐dependent competition during the crown closure stage in forest stand development; and trees with larger crowns are more likely to produce more offspring over their lifetimes. Biotic and abiotic stresses will reduce growth rates. Height growth increment and height growth rate per day have moderate to high population differentiation (*Q*_ST_), on average (Alberto et al. 2013), suggesting population variation in growth relates to local adaptation. Forest trees with wide ranges and continuous distributions generally have lower *F*_ST_ estimates and higher *Q*_ST_ estimates than species with smaller or fragmented ranges (Alberto et al. 2013).

### Cold and drought hardiness

Cold and drought hardiness are important traits reflecting adaptation to abiotic stresses, but they are difficult to assess in field tests, as damage from extreme weather events occurs sporadically, and injury or mortality can be difficult to attribute to the correct agent. Methods have been developed to phenotype seedlings for cold hardiness, drought hardiness and phenology in common garden experiments in controlled nursery or growth chamber environments (St Clair and Howe 2007; K.J. Liepe, A. Hamann, P. Smets, C.R. Fitzpatrick and S.N. Aitken, unpublished data). Methods have also been developed to screen for resistance to specific insects and diseases, but these are host and pest specific and beyond the scope of this review.

**Figure 2:**
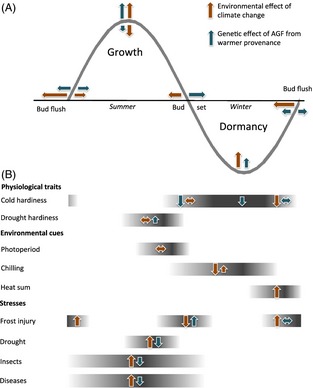
(A) A schematic diagram of the annual developmental cycle of temperate and boreal trees for 1 year illustrating the timing of growth, dormancy and bud phenology, modelled after the degree growth stage model of Fuchigami et al. (1982). (B) The timing of physiological changes, environmental cues, and biotic and abiotic stresses. Orange arrows illustrate the expected environmental effects of climate change (timing, magnitude and direction); blue arrows indicate the expected genetic changes in phenotypic traits and vulnerability resulting from assisted gene flow from populations matching projected new conditions.

To be well adapted to local temperatures, trees need to be able to utilize the available frost‐free period and grow competitively as long as other environmental conditions remain favourable for growth, yet cease growth and develop cold hardiness in response to lengthening nights before damaging fall frosts occur (Fig. 2). In a warming world, cold hardiness may remain an important trait as disruptions to seasonal thermal cues can result in unseasonal growth, especially in late winter and spring (Harrington and Gould 2015). Fall cold hardiness and bud set phenology show strong population differentiation and strong clines along provenance thermal gradients, and less phenotypic plasticity than spring events (Howe et al. 2004; Savolainen et al. 2007; Alberto et al. 2013). Height growth cessation and bud set in temperate and boreal trees can be triggered by photoperiod, temperature cues or endogenous factors, depending on the species (Cooke et al. 2012), and drought or other stresses can result in earlier cessation (Howe et al. 2004). Winter and spring cold hardiness and bud flush phenology show weaker and more variable patterns of variation among populations and show strong plasticity in response to variation in chilling and heat sum accumulation (Duputié et al. 2015; Harrington and Gould 2015). Provenance variation in growth often reflects a direct tradeoff with cold hardiness and frost‐free growing season length (Langlet 1971; Howe et al. 2004; Savolainen et al. 2007). The development of methods for artificial freeze testing of large numbers of detached shoot or leaf samples from common garden experiments has improved phenotyping of cold hardiness (e.g. Aitken and Adams 1996; Hannerz et al. 1999). Not all phenological clines necessarily evolve from avoidance of cold injury: Soularue and Kremer (2014) showed through population simulations that non‐adaptive clines in reproductive phenology can evolve along environmental gradients as a result of selection for overlapping flowering periods in populations in different environments.

Clines in growth rate in provenance trials are often more strongly correlated with provenance temperature regimes than with annual or seasonal precipitation variables for many temperate and boreal species (Rehfeldt et al. 1999; St Clair et al. 2005; Wang et al. 2006; K.J. Liepe, A. Hamann, P. Smets, C.R. Fitzpatrick and S.N. Aitken, unpublished data) (also see ‘Shared patterns of adaptive variation’ section below). While interspecific variation in drought tolerance is high, drought‐related phenotypic traits generally show weaker population differentiation than temperature‐related traits (McDowell et al. 2008; Alberto et al. 2013). Drought hardiness has become a greater concern than it was historically, as climate change is having uncertain and variable effects on precipitation patterns, and drought‐related mortality in natural forests due to hydraulic failure or carbon starvation has increased (McDowell et al. 2008; Allen et al. 2010; Chmura et al. 2011). As rising temperatures create greater evapotranspirational demands, traits related to water use are also receiving more attention. However, drought hardiness is challenging to phenotype due to the complexity of responses to low water availability, including physiological and morphological mechanisms of drought avoidance or tolerance, and potential phenotypic effects of drought including growth reductions, injury or mortality. Many species are quite drought tolerant later in the growing season and acclimate following drought exposure (Kozlowski and Pallardy 2002), but exposure to drought earlier can result in injury or premature growth cessation. It is not clear the extent to which greater water‐use efficiency due to higher CO_2_ concentrations will compensate for temperature‐related increases in drought stress (Chmura et al. 2011). Finally, predictions for changes to precipitation regimes from global circulation models vary more than temperature predictions, and so greater uncertainty exists around future water availability than future temperatures. Planting forests with high genetic diversity, including seedlings from warmer, drier populations, should provide some buffering against this uncertainty as long as sites remain within a species’ bioclimatic niche.

### Population variation in adaptive and neutral genetic markers

While genecological studies suggest divergent selection on phenotypic traits for local adaptation to climate must be relatively strong (Howe et al. 2004; Savolainen et al. 2007; Alberto et al. 2013), population genetic studies suggest gene flow is high, as most widespread species show weak‐to‐moderate population differentiation (*F*_ST_) for selectively neutral genetic markers (Kremer et al. 2012). How tree populations could diverge substantially for locally adaptive traits in the face of high levels of gene flow has been something of a puzzle (Savolainen et al. 2007). Theoretical modelling suggests that highly polygenic traits controlled by many co‐varying loci of small effect can create phenotypic divergence under divergent selection despite high gene flow, but individual loci underlying such traits will have only weak divergence, and will be difficult to detect (Latta 2003; Le Corre and Kremer 2012; Savolainen et al. 2013). This genetic architecture presents a challenge for population studies to detect and adequately characterize local adaptation through genome scans.

The genetic architecture of quantitative traits that show strong population divergence in population genomic studies seems to fit this expectation of many loci, each with small effect (Alberto et al. 2013). Quantitative trait loci mapping approaches (Jermstad et al. 2001a,b) and genomewide association studies (e.g. Gonzalez‐Martinez et al. 2007, 2008; Eckert et al. 2009; Holliday et al. 2010a; Evans et al. 2014) have found multiple loci, each with small effect sizes, for traits including growth, cold hardiness, bud phenology and water use.

**Figure 3:**
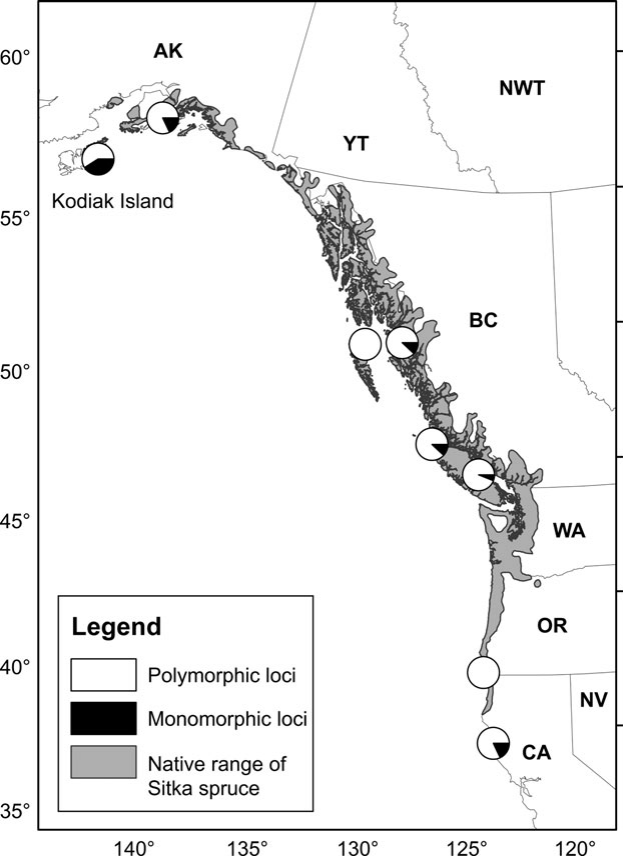
Map of *Picea sitchensis* showing the proportion of 17 putatively adaptive SNPs found to be associated with fall cold hardiness by Holliday et al. (2010a) that were polymorphic based on genotyping of 122 to 164 mature trees in each population, from Lobo (2011).

As geographic patterns of variation are weaker for individual adaptive loci than for the quantitative traits they underlie, high levels of variation can be maintained within populations at these adaptive loci. For example, Lobo (2011) genotyped mature trees in each of eight populations across the range of *Picea sitchensis*, including continuous and disjunct populations, for 17 putatively adaptive SNPs previously associated with cold‐hardiness phenotypes showing strong population differentiation in a genomewide association study (Holliday et al. 2010a). Comparatively large sample sizes (*N* = 122–164 trees per population) allowed for the detection of standing variation in the form of adaptive alleles present at low frequency, and the genotyping of older trees allowed assessment of adaptive alleles present in reproductively mature populations. Most of these SNPs showed clinal variation in frequencies across the species range, but were polymorphic in most populations (Fig. 3), with ‘warm’ alleles (those associated with lower cold hardiness or later bud set timing and at high frequency in warm populations) present at low frequency in cold populations, and ‘cold’ alleles present in warm populations at low frequencies. The only population that was markedly genetically depauperate was the recently founded and inbred Kodiak population, thought to have colonized the east end of the island about 500 years ago (Mimura and Aitken 2007a,b; Holliday et al. 2010b). If AGF were implemented in this species using individuals from warmer populations for reforestation in cooler locations, it would be expected to change allele frequencies and increase the number of pre‐adapted genotypes with adapted combinations of alleles, but would not introduce many alleles that are not already present at low frequency.

Population genomic approaches that analyse large numbers of loci have the potential to provide genetic information on the environmental drivers and spatial scale of local adaptation within a much shorter timeframe than provenance trials. Sampling designs and analytical methods for detecting outlier loci under divergent selection through genotype–environment associations are maturing (e.g. Günther and Coop 2013; Lotterhos and Whitlock 2015). In a large‐scale population genomics project of two widespread conifers in western Canada, genotype–environment association tests have identified the same climatic drivers and patterns of local adaptation as short‐term seedling experiments in lodgepole pine (*Pinus contorta*) and in the interior spruce complex (*Picea glauca*,* P. engelmannii* and their hybrids) (S. Yeaman, K.E. Lotterhos, K.A. Hodgins, K.A. Nurkowski, L.H. Rieseberg, M.C. Whitlock and S.N. Aitken, unpublished data). However, in this synthesis we focus on phenotypic variation in common garden experiments for characterizing patterns of local adaptation as few comprehensive population genomic studies are available at this time.

## Using common gardens to predict responses to climate change

The use of provenance variation to adapt tree populations to climate change is not a new idea. Over 20 years ago, when the threat of climate change to biodiversity was emerging, Ledig and Kitzmiller (1992) were the first to recognize the potential for AGF:

‘If global warming materializes as projected, natural or artificial regeneration of forests with local seed sources will become increasingly difficult. However, global warming is far from a certainty and predictions of its magnitude and timing vary at least twofold. In the face of such uncertainty, reforestation strategies should emphasize conservation, diversification, and broader deployment of species, seed sources, and families. Planting programs may have to deploy non‐local seed sources, imported from further south or from lower elevations, which necessitates a system for conserving native gene pools in seed banks or clone banks’.

Mátyás (1994) anticipated the value of provenance trials as climate change experiments that could predict evolutionary responses to climate change and identify pre‐adapted provenances for future climates, by substituting spatial climatic variation for temporal variation (although provenance trials have some limitations; Aitken et al. 2008; Franks et al. 2014). Mátyás also argued for selecting and breeding genotypes that performed well over a wide range of climates, and investigating what genetic mechanisms produced phenotypes with broad climatic stability and consistent productivity. Twenty years later, we still have little idea why some provenances are productive over a wide range of climates, while others have a relatively narrow productive niche (e.g. Wang et al. 2006).

The first comprehensive analysis of provenance trial data for predicting the response of populations to climate was by Rehfeldt et al. (1999). They analysed 20‐year growth data from the Illingworth lodgepole pine provenance trial in British Columbia, an incomplete reciprocal transplant experiment with 118 provenances and 60 test sites. Response functions describing individual population growth as a function of individual climatic variables at test sites were fit using a quadratic regression approach. It is worth noting that 45% of the response functions were not significant at *P* < 0.1, indicating that local adaptation to climate is not always detectable, and that there is a lot of unexplained site‐to‐site environmental variation in performance at a given temperature or moisture regime (also see supp. figures in this study). Wang et al. (2006) re‐analysed the Illingworth trial using improved climatic data and a new approach for anchoring response functions of populations based on extrapolations of niche margins from transfer functions. Better matching of lodgepole pine populations to new climates was projected to yield 10–35% more wood than *status quo* local seed use as in the shorter term, growth was projected to increase over historic levels due to warming of cold‐limited environments, but then decrease farther into the future as warmer sites become more heat and drought limited. The same data were used by Wang et al. (2010) to develop the universal response function (URF) approach, integrating the climatic effects of provenance and planting site in a single model, and to facilitate selection of the best seed sources for projected future climates for a given planting environment. While comprehensive field provenance trials can provide excellent knowledge for designing species‐specific AGF strategies, few species have such comprehensive studies for scientists to draw on, and much data on provenance variation is from a single nursery or field test environment, or from sites that are not warm enough to serve as proxies for future climates.

## Shared patterns of adaptive variation in western North American species

To what extent are patterns of local adaptation along climatic gradients similar among species within a given geographic region? If clines in phenotypic traits are similar, can average patterns of sympatric variation guide AGF or identify critical climatic variables as a first approximation for untested species? We reanalysed data from the literature on provenance trials in temperate tree species from western North America to address these questions. This is an ideal region of focus because of the many provenance trials conducted on native tree species that are important for forestry, and because its high topographic complexity means that turnover in climate occurs rapidly and heterogeneously over short geographic spaces. Clines along climate gradients are thus especially likely to reflect local adaptation to climate, rather than non‐adaptive phenomena resulting from population demographic history.

### Provenance trial data sets

We focused on adaptive clines in three traits expected to impact fitness of natural populations that are commonly phenotyped: height growth potential, timing of spring shoot phenological events associated with growth initiation (‘spring events’), and timing of fall phenological events associated with growth cessation (‘fall events’). We identified clines along gradients in mean annual temperature (MAT), selected as a broad proxy for overall climate, and mean summer precipitation (MSP), intended to reflect growing season drought stress. Due to few studies on trees species from very dry climates, and less consistent adaptive clines identified along the gradient in MSP, we focus primarily on results for MAT and caution that our results may not be applicable to species and regions where climates are becoming much drier and drought‐related mortality is a major concern (Allen et al. 2010).

We searched the literature for data sets from provenance trials in which height or timing of spring or fall shoot events was reported. Our search was restricted to temperate tree species native to western Canada and the United States, west of and including the Rocky Mountains. Species native to this region but found primarily in boreal forests, deserts, grasslands or Mediterranean climates were excluded. We initially searched using *Web of Science* (Thompson Reuters, New York, NY) for articles on provenance trials and common gardens individually for each tree species native to British Columbia (Klinka et al. 2000). We then expanded this search to include all conifers and common angiosperm trees (Little 1971) that met our broader geographic and ecological criteria, by scanning titles of search results for species plus the word ‘provenance’ in the University of Michigan Library (using *Summon* ^®^, ProQuest, Ann Arbor, MI, USA). We retained data sets in which multiple populations were grown in a common environment, including greenhouse environments and field sites within and beyond the species’ native range. All data sets for ‘spring events’ recorded the timing of bud flush, whereas we pooled timing of bud set, growth cessation and leaf abscission into a single category of ‘fall events’ because of an insufficient number of studies assessing bud set. All three fall events are steps towards the initiation of dormancy prior to development of cold hardiness, and the timing of these events reflects local adaptation to maximize the length of the growing season while avoiding fall frost injury (Howe et al. 2004; Rohde and Bhalerao 2007; Cooke et al. 2012). In natural populations of *Populus*, the timings of these events are highly correlated (Rohde et al. 2011, McKown et al. 2013). Although genetic mechanisms underlying these traits are complex (Cooke et al. 2012), pooling fall events is justified given the shared pressures from natural selection that underlie observed patterns in all three traits.

We applied four additional filters to control for quality and ensure comparability of data sets among species: (i) the common environment must have been free of major pest or pathogen outbreaks, severe frost or drought damage, and other biotic or abiotic stressors. These stressors could differentially impact certain genotypes and thus mask genetic differences in growth potential among populations. (ii) Provenance geographic coordinates must have spanned at least five degrees of latitude or 50% of the species distribution to ensure clines reflect broad‐scale species‐level patterns. Small, geographically isolated populations and populations from a different subspecies or variety compared to the rest of the other populations were also excluded. (iii) To avoid pseudoreplication, if height data were available for the same set of populations tested at multiple sites or measured at different ages, only the most recent data from the site with the greatest mean height were included. Because height was assessed at locations with relatively high site productivity, clines represent height growth potential under good growing conditions. If multiple observations of spring or fall events were available, only those from the year with the greatest variance in timing of these events were included. (iv) Studies must have met minimum sample size criteria. These criteria recognize the practical limitations to experiments in total sample size and tradeoffs between the number of individuals per population and the number of populations for estimating clines. While experiments with many individuals per population (bulk samples) or more families per population estimate means precisely, experiments with many populations provide better estimates of clines. Thus, we accepted studies that had (i) at least 50 populations with as few as three individuals each, (ii) at least nine populations of at least three open‐pollinated families each with at least three individuals per family, or (iii) at least nine populations with 25 individuals each, regardless of family structure. Populations and open‐pollinated families not meeting these criteria were excluded, if population size and family structure were known (Tables S1–S3). Timing of spring and fall events must have been measured in units of calendar days, not using a quantitative scale on a single day.

**Figure 4:**
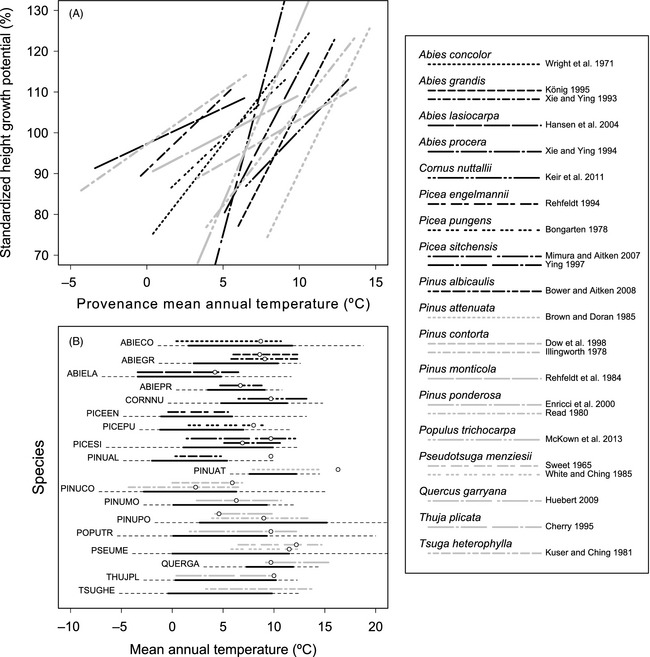
Adaptive clines in height growth potential along a temperature gradient are similar among many temperate tree species from western North America. (A) Regression slopes of standardized height growth potential versus provenance mean annual temperature (MAT), for populations grown in a common test environment. Each line represents an independent provenance trial, and line type corresponds to the species and reference indicated in the legend. Only statistically significant regression slopes are shown (*P* < 0.05). (B) The climatic scope of provenance trial data sets compared to the species realized niche. For each species, the bottom line represents the core climatic niche (solid black line) and full climatic niche (dashed extension) in MAT (see text; Thompson et al. 2000). The upper lines represent the range of provenance MAT values among populations within each provenance trial for that species. Open circles represent the MAT at the test site(s) for each provenance trial. Provenance trials missing an open circle were grown in a controlled environment. Note that the *y*‐axis scale in (A) and *x*‐axis scale in (B) do not cover the full range of data for some species; this scaling was applied to increase visibility for the majority of the data sets.

Provenance MAT was estimated for each population using *ClimateWNA* (Wang et al. 2012) with provenance latitude, longitude and elevation provided in original publications. Simple linear regressions of population mean values of phenotypic traits versus provenance MAT were performed for each species (Fig. 4A). To facilitate comparisons among species, regression slopes for height growth potential were rescaled to units of percent change per degree Celsius increase in provenance MAT (%·°C^−1^), with 100% assigned to the expected height growth potential at the midpoint of provenance MAT. Regression slopes for timing of spring and fall events were retained in days per degree Celsius increase in provenance MAT (d·°C^−1^), but a value of zero was assigned to the expected date of spring and fall events at the midpoint of the provenance MAT values. Similar analyses were conducted for population MSP (Tables S1–S3). Although variation in phenotypic traits along climatic gradients may be nonlinear and better modelled by a quadratic function, visual inspection confirmed that most relationships were approximately linear (Figures S2–S9), and linear models were applied to all species to facilitate interspecific comparisons.

A total of 23 data sets (for 18 species) were available for height growth potential, 11 data sets (eight species) for spring events and six data sets (five species) for fall events (Fig. 4B, Tables S1–S3). Datasets containing population means from all studies are archived in Dryad (Aitken and Bemmels 2015). In addition to documenting adaptive clines, we determined how representative the provenance trial data sets are of climatic conditions found across each species’ geographic range. To assess this, we compared the range of MAT and mean July precipitation (MJP) among provenances to the realized niche of each species along gradients in the same climate variables. Species realized niches were obtained from Thompson et al. (2000), who calculated niches based on the presence–absence data over a 25 by 25 km grid of North America. MJP estimates were used to evaluate breadth of precipitation among provenances as Thompson et al. (2000) did not estimate MSP. The ‘full’ climatic niche of the species corresponding to 100% of the presence observations is likely overestimated due to climatic outliers induced by scaling and methodological error, whereas the ‘core’ climatic niche represented by the middle 80% of the presence observations may be a more realistic estimate of niche (Thompson et al. 2000). Both the full and core climatic niches for each species were compared with the climatic scope of each provenance trial (Fig. 4B, Figure S1B). For two species (*Cornus nuttallii* and *Populus trichocarpa*) that were not included in the initial data set (Thompson et al. 2000), we estimated climatic niches ourselves: we converted species distribution maps (Little 1971, 1976) to the presence–absence data on a 25 by 25 km grid and extracted climate data (10‐arcminute resolution; (White 1987; Hijmans et al. 2005)) from the geographic centre of each grid cell in which the species was present.

### Strong, shared clines in growth potential along a temperature gradient

Regressions of population mean height growth potential on provenance MAT were statistically significant (*P* < 0.05) in 14 of 23 data sets (13 of 18 species; Fig. 4, Table S1). Population mean height growth potential increased by an average of 3.6% per degree Celsius increase in provenance MAT, which statistically differed from zero (*t*‐test: *P* < 0.0001, 95% confidence interval 2.1 to 5.1%·°C^−1^, non‐significant regressions included). Mean *R* ^2^ was 0.43 (range: 0.19 to 0.89, significant regressions only). The relationship between height growth potential and provenance MAT was positive in all statistically significant cases. It is remarkable that with such a simple analysis and such a broad proxy for overall climate, adaptive clines of similar magnitude and consistent direction were detected in so many species. There is undoubtedly much nuance to patterns of adaptation not captured by our results. For example, genotype‐by‐environment interaction, including the impacts of damage due to cold or drought, may create adaptive clines that are nonlinear, or vary in magnitude or even direction depending on the harshness of the test site (White 1987; Ying 1997; Porter et al. 2013). Our linear clines from data collected in relatively warm but productive sites, on average, could be considered indicators of growth potential of provenances, rather than predictors of growth on all sites. Differentiation may also be associated with other climatic and non‐climatic factors. Despite these nuances, our consistent findings suggest that local adaptation to climate is common and that general clines in height growth potential along a temperature gradient in this region are shared among many species. While these analyses are simple and may not detect fine‐scale patterns of variation associated with other environmental gradients, most seed zones and seed transfer guidelines for reforestation do not currently address nuanced patterns of adaptation either, but rather delineate broad map‐based areas within which seed can be moved freely or within elevational limits. Developing highly complex models to incorporate all potential sources of variation for all species into management recommendations is not feasible without extensive data, operationally intractable to implement in reforestation or restoration, and unnecessary given high within‐population variation.

**Figure 5:**
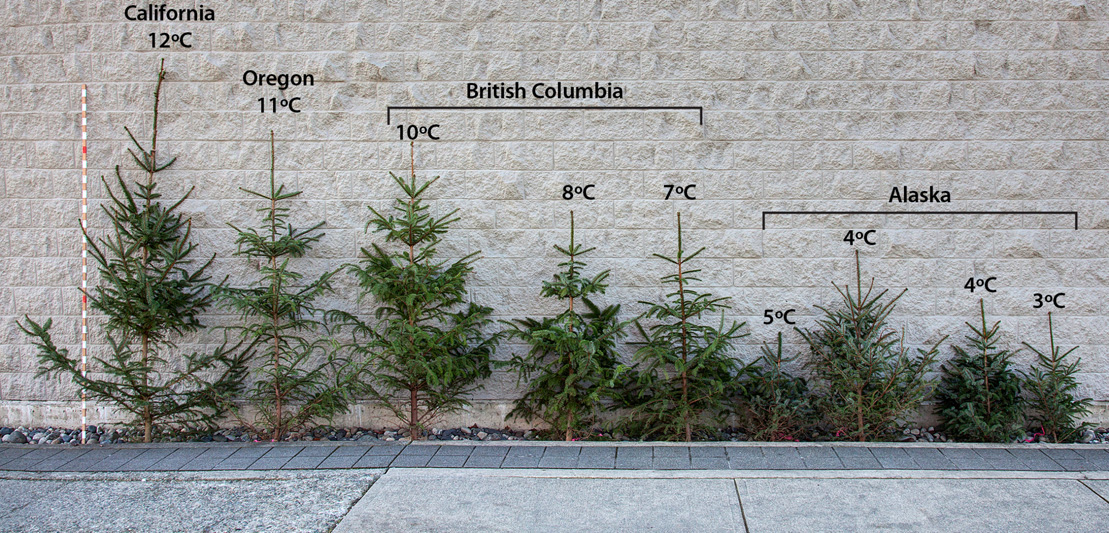
Provenance variation in 8‐year‐old *Picea sitchensis* from across the species range grown in a common garden in Vancouver, BC, Canada. For illustrative purposes, trees were selected from each population that were closest to the provenance mean. The state or province of origin and the provenance mean annual temperature are indicated. This species shows the steepest cline of all species for height at age 2 in Figure 4(A). The population from Kodiak Island, AK (Fig. 3) has poorer growth than expected for an MAT of 5°C, likely due to inbreeding (Mimura and Aitken 2007b).

The absence of a cline in height growth potential with MAT in five species does not necessarily mean that they are not locally adapted. Height growth potential was significantly correlated with provenance MSP in *Pinus monticola* (*P* < 0.0001, Figure S5) (Rehfeldt et al. 1984)), and the pattern was marginally non‐significant in *Pseudotsuga menziesii* (*P* = 0.070, (Sweet 1965)) and *Quercus garryana* (*P* = 0.055, (Huebert 2009)). These results suggest that adaptation to drought may be more apparent in certain species than adaptation to temperature or that temperature‐related adaptation may be expressed in traits other than height growth potential. For *Pinus albicaulis*, Bower and Aitken (2008) report higher *Q*_ST_ in traits related to cold adaptation than in growth traits, not surprising for an extremely slow‐growing species found at the upper treeline in harsh subalpine climates. Only in *Abies procera* (Xie and Ying 1994), a species with a relatively narrow and discontinuous climatic distribution, did our reanalyses detect no evidence of adaptation, although traits that were not analysed may be locally adapted. At the other extreme, the steepest cline in growth potential was for *Picea sitchensis,* a species with a long, narrow distribution along the Pacific coast and strong local adaptation (Fig. 5).

Local adaptation appears to be extremely common in widespread tree species and more common than in plants in general, although we have not used stringent reciprocal transplant criteria to define local adaptation. Leimu and Fischer (2008) found evidence of reciprocal local adaptation *sensu stricto* in plants in only 45% of pairwise population comparisons and superior performance of local genotypes in 71% of test sites. They also found that species with large population sizes, true for most widespread tree species, are more likely to be locally adapted. While our criteria are less stringent for detecting local adaptation, they are more risk averse in terms of forest management and conservation, as it is safer to assume that populations differ when they do not than to assume that they are not locally adapted when they are.

### Clines in shoot phenology are more consistent in fall than spring

Statistically significant regressions of timing of spring events versus provenance MAT were found in six of 11 data sets (four of eight species; Table S2). The signs of slopes varied and were mostly shallow, ranging from −5.1 to 4.6 d·°C^−1^, with a mean *R* ^2^ of 0.46 (range: 0.26 to 0.82; significant regressions only). In three species, spring events occurred earliest in populations from colder provenances, while in one species, the opposite trend was observed. In contrast, regressions of timing of fall events versus provenance MAT were statistically significant and consistent in direction in all six data sets (five species), with fall events occurring earliest in populations from colder provenances (Table S3). Population mean date of fall events increased by an average of 6.3 d·°C^−1^ (range: 0.89 to 11.4 d·°C^−1^; t‐test for significant difference from zero: *P* = 0.0149), with a mean *R* ^2^ of 0.63 (range: 0.25 to 0.90). Alberto et al. (2013) also concluded elevational and latitudinal clines in bud set were more consistent among species than clines in bud flush in a larger number of temperate and boreal tree species from multiple continents.

Weak or inconsistent clines in spring events, in contrast to strong, consistent clines in fall events, may reflect more complex patterns of local adaptation in spring. While fall events are usually triggered by critical photoperiod, or in some cases by completion of predetermined growth, spring bud flush is initiated after attaining both sufficient chilling and an adequate heat sum (Fig. 2). The average timing of bud flush for a population may depend on particular local climatic patterns that allow for adequate chilling and heat sum accumulation at the transition from winter to spring. Chilling and heat sum requirements, and the timing of bud flush in a given environment, can vary among sympatric species; for example, opposite directions of adaptive clines in bud flush have been reported along a shared elevational gradient in *Fagus sylvatica* compared to *Fraxinus excelsior* and *Quercus petraea* (Vitasse et al. 2010).

Complex and species‐specific adaptive variation in bud flush timing would render the impacts of climate change and AGF much less straightforward for spring than for fall events or height growth potential. While climate change is increasing heat sum accumulation in the spring, its effects on chilling are mixed. In many locations, the occurrence of effective chilling conditions (between 0 and 5°C) is decreasing, but in some cold locations with common subfreezing temperatures, chilling is increasing with warming (Harrington and Gould 2015). Chilling and heat sum accumulation can occur simultaneously to some extent. Harrington and Gould (2015) predicted the effects of climate change on bud flush timing in 11 tree species in the US Pacific Northwest and concluded that most species in most locations would have far earlier bud flush with winter warming of 3 to 5°C, with only a small rear‐edge portion of the range of a few species not receiving sufficient chilling and having delayed bud flush due to environmental effects. The genetic variation we observed in our analyses is far outweighed by this environmental variation. The potential for phenotypic plasticity in bud flush timing as a response to climate change is high, and this phenotypic plasticity is not always adaptive (Duputié et al. 2015). Even if a genetic mismatch were introduced between realized and optimal timing of bud flush under future climates through AGF, this mismatch would likely be minor.

### Knowledge of adaptive clines is extensive

The climatic scope of the 23 data sets we reanalysed for height growth potential covers a large proportion of each species’ realized niche in temperature (Fig. 4B) and precipitation (Figure S1B). Provenances sampled spanned on average 72% of the core climatic niche in MAT and 87% in MJP. A number of biases were evident in the data sets: (i) species composition is heavily biased towards commercially important conifers. (ii) The climatic scope of data sets is biased towards the warmer and wetter portions of species ranges (average difference between midpoint of provenances and midpoint of species core range: MAT = 1.4°C, *P* = 0.0003; MJP = 7.8 mm, *P* = 0.0511). This pattern likely reflects greater interest of foresters in faster‐growing populations from milder environments, and greater sampling accessibility of these populations, and these are also areas where forest harvest and reforestation is most likely to occur. (iii) Test sites for common gardens were also warmer and wetter than average (average difference between test site and midpoint of species core range: MAT = 2.9°C, *P* = 0.0001; MJP = 20.8 mm, *P* = 0.0349). This may also reflect our deliberate avoidance of data sets for experiments with major damage due to biotic or abiotic stresses that could differentially inhibit growth potential among populations.

Despite these biases, the breadth of knowledge revealed from provenance trials in western North American tree species is large, and our analysis includes only a subset of these trials. Our results are consistent with studies including other species and regions (Howe et al. 2004; Savolainen et al. 2007; Alberto et al. 2013). Overall, (i) the number of species studied, (ii) the climatic breadth included in provenance trials, (iii) comparisons with analyses in other regions of the world, and (iv) the consistent finding of strong, shared adaptive clines in height growth potential and fall events support the conclusions that local adaptation is common in widespread temperate trees, and that patterns of adaptation along climatic gradients are often very similar among species. For sympatric species in which costly provenance trials have not been conducted and specific knowledge of local adaptation is lacking, the average patterns of adaptive variation in tree species from this region are likely to serve as a reasonable approximation of adaptive clines. These average patterns can be used for proceeding with AGF in untested temperate species, with high potential benefits and low risk of introducing genetic maladaptation to populations. These patterns can also be used to make genetic decisions for assisted species migration (Fig. 1), but because of the ecological effects of species introductions, we limit our discussion here to AGF within existing species ranges.

## Recommendations for AGF for forest trees

It is time to change reforestation policies and practices from an emphasis on local seed sources to a framework for AGF in widespread temperate and boreal tree species. These changes are under consideration in some jurisdictions in North America and in Europe, but many others are waiting for more information before action, a delay syndrome common to most climate change adaptations. For all such species, we recommend that seed zones and seed transfer rules be adjusted so genotypes that are pre‐adapted to near‐future conditions are used for reforestation and restoration, either alone, or with composite provenancing in intimate mixtures with more local seed sources (Broadhurst et al. 2008). Here, we consider how to best mitigate risks and capture benefits of AGF.

### Avoiding disruption of seasonal cycles of growth and dormancy

The risks of AGF need to be evaluated against *status quo* local seed use in projected future climates. We have shown above that most populations from warmer provenances compared with cooler provenances consistently have greater growth potential over a longer growing season, set bud and develop cold hardiness later, and may burst bud earlier or later (Fig. 2). Seedlings moved from milder to cooler locations through AGF should better match new temperature and moisture regimes than local populations, enabling them to better utilize the available growing season.

Climate warming could cause extreme disruption of growth cycles in local populations in some limited cases for environmental rather than genetic reasons. Relationships between chilling and heat sum requirements are complex. Some extreme southern and coastal populations of some species may no longer receive sufficient chilling to break bud and resume growth normally, while others may meet chilling sums earlier due to an increase in days with temperatures just above rather than below freezing and effective for chilling (Harrington and Gould 2015). Some species that achieve necessary heat sums for bud flush very early in spring may detect their critical night length well before the summer solstice, resulting in premature growth cessation, a phenomenon observed in *Populus balsamifera* transferred large distances south (Soolanayakanahally et al. 2013). However, if these extreme disruptions occur in local populations, they are unlikely to be exacerbated by AGF from warmer to cooler locations, and may result in areas moving outside of a species’ bioclimatic niche space. Bioclimatic niche models should be used to determine whether an area is likely no longer within the climatic niche space of a species.

Research on individual species has shown that populations from drier environments can have greater drought hardiness (e.g. White 1987), although population differentiation is relatively weak for most water use and drought‐related traits (Alberto et al. 2013). We recognize that our analysis does not directly address local adaptation to drought; however, it is interesting that phenotypic relationships between growth and MSP are non‐significant or negative in species except *Pinus ponderosa* and *Picea pungens*, where growth is positively correlated with MSP, and *Pinus monticola*, where the California provenances have low growth potential (Figure S1A, Tables S1–S3). Moderate drought stress can reduce growth, while severe drought stress can result in tree mortality through hydraulic failure or carbon starvation (McDowell et al. 2008; Sala et al. 2012).

Drought is likely to be a major driver of range contraction at species rear range margins, and bioclimatic modelling should identify geographic areas becoming too dry for any provenances of a species. It will be important to prioritize such provenances as sources for AGF to suitable habitats expected to persist within the species climatic niche, and for *ex situ* conservation. In other parts of a species range remaining within the species bioclimatic niche but becoming warmer and drier, AGF of seedlings from warmer, drier environments should be better adapted to planting climates than local seedlings. We caution against moving seedlings from warmer, wetter locations to drier locations, although this risk will depend on the seasonal timing of these stresses as seedlings can tolerate considerable drought stress late in the growing season (Kozlowski and Pallardy 2002; McDowell et al. 2008). There is considerably more uncertainty around precipitation than around future temperature regimes in downscaled climate change projections. Assisted gene flow strategies should strive to locate source populations that match both temperature and moisture regimes, where possible, and managers should consider composite provenancing with more than one source population to increase diversity on drought‐prone sites. Species‐ and population‐specific research on variation in temperature and drought hardiness can be used to refine initial AGF strategies over time.

### Climate projections and patterns of provenance variation for prioritizing source populations and sites for AGF

The projected rate of climate change varies geographically, for example with higher latitudes warming more rapidly than lower latitudes. Assisted gene flow strategies should be designed to re‐match genotypes with projected climatic habitats in the near future. Depending on the information available, AGF strategies and policies can be designed based on: (i) climate models alone, (ii) climate models combined with patterns of local adaptation identifying climatic drivers of differentiation for sympatric species, or (iii) climate models combined with species‐specific provenance trial data. Gray and Hamann (2013) used climatic data in their analysis of historic, current and future distributions of population‐specific bioclimate envelopes of 15 tree species from western North America, assuming major ecosystem units were a reasonable proxy for populations. They concluded that populations already lag an average of 130 km in latitude or 60‐m elevation behind their climatic niches, assuming populations occupied their optimal climates prior to anthropogenic warming. They forecast that this lag would increase to 340 km in latitude or 140 m in elevation by the 2020s. While estimated lags varied somewhat among populations within species, and among species within regions, they were generally of similar magnitude and always in the same direction. Given the rapidity with which these climate change‐induced adaptational lags are growing over time, using generalized patterns of provenance responses or bioclimatic envelop shifts seems to carry less risks than continuing with local seed use. In some cases, projected near‐future climates will have no analogues in the 20th century climate normal. In this event, both species and genetic diversity should be increased to buffer against uncertainty.

At any given location, the horizontal movement required to track a given climate spatially depends on both the local rate of climate change and the topographic heterogeneity, as climate changes more rapidly with increasing elevation than with increasing latitude or longitude. Loarie et al. (2009) developed the concept of velocity of climate change, an estimate of distance/time required to spatially track climate. Their method did not take into account that movements upslope end in cul‐de‐sacs once mountaintops are reached. Hamann et al. (2014) developed new algorithms to take such discontinuities into account. They produced maps of ‘forward’ and ‘reverse’ velocities and vectors of climate change that can be used to rank populations and sites for AGF.

Reverse velocities (future‐to‐present) can be used to evaluate planting sites that will benefit most from AGF, for example in terms of health, productivity and carbon sequestration. In general, valley bottom sites and large, relatively flat areas will likely benefit the most from AGF as they have the highest velocities and are farthest from pre‐adapted populations.

Forward velocities (present‐to‐future) indicate the distance and direction that populations would need to move in order to track climate. Higher forward velocities indicate populations that are the farthest from suitable future habitat. These populations should be high priority for both *ex situ* conservation, and for AGF moving individuals to a location where they are more likely to persist as climates warm. Generally, mountaintops have the highest forward velocities as populations in these areas cannot migrate uphill farther and would have to travel large horizontal distances to find suitable future climates.

Local topographical variation can affect levels of genetic variation within populations due to gene flow across heterogeneous landscapes. Yeaman and Jarvis (2006) found lodgepole pine populations in mountainous areas of high topographic variability had greater quantitative genetic variation for phenotypic traits those in flatter landscapes. This suggests that in addition to higher velocities, populations in less topographically variable areas may have less genetic variation for evolutionary responses to climate change, and may benefit most from AGF.

### Extent of adaptational lag

Historic seed transfer policies generally assumed that local populations in different parts of a species range were equally well adapted to their local climates. However, many widespread, continuously distributed tree species likely have abundant centre distributions, with higher population densities in the centre of the range than at the peripheries. If this is the case, gene flow is predicted to be asymmetrical and greater from central populations to the periphery than the reverse (Kirkpatrick and Barton 1997), as estimated for *Picea sitchensis* based on single nucleotide polymorphisms (Holliday et al. 2012). For a species distributed along a temperature gradient, for example latitudinal or elevational, this means populations originating from near the warmer species margin should be adapted to cooler conditions, on average, than they inhabited historically, and those towards the colder species margin should be adapted to warmer conditions. Lodgepole pine in British Columbia shows evidence of this pattern in northern provenances (Rehfeldt et al. 1999; Wang et al. 2006). The degree of maladaptation resulting from climate change will be greater for populations from the warmest portion of the species range, while those in the colder portion of the range may benefit from slightly warmer temperatures *in situ*, and are at less risk of maladaptation in the short term (Davis and Shaw 2001; Davis et al. 2005). For species with abundant centre distributions, populations towards the warmer species margins, but expected to still be within the species climatic niche, and central populations will benefit the most from AGF, with those towards the cooler margin still benefiting but to a lesser extent. The different slopes of some species in Fig. 4A may reflect varying effects of gene flow homogenizing populations, rather than different selection pressures.

### Time frame to target for AGF

Climate change presents a moving target for matching genotypes to sites. For short‐rotation length woody crops such as poplars and *Eucalyptus*, this is not a big problem, but for the majority of temperate species with rotation lengths ranging from 40 to 100 years, it is challenging to determine what life stage AGF should target. The first few years of a seedling's life are the most vulnerable to abiotic stress, for example cold injury and drought stress, but wood production and carbon sequestration rates are highest around mid‐rotation. Trees that might be well adapted to conditions later in the rotation, but that do not grow well under conditions early in the rotation, are likely to die due to density‐dependent competition and not become part of the overstory. We recommend that if a single seed source is used for reforestation, that it be matched for temperature regime, and if possible for moisture regime, with expected site conditions in about two decades. If composite provenancing of multiple sources is used, we recommend combining seedlings from a source population where climate matches current conditions with seedlings from a source where climate matches predicted conditions in approximately two decades.

### Genetic diversity

For widespread temperate and boreal species, especially those that are wind‐pollinated (e.g. all conifers), genetic diversity for selectively neutral genetic markers diminishes somewhat across species ranges from central to peripheral populations, on average (Hampe and Petit 2005), but this effect is generally very weak in widespread species with continuous ranges (S. Aitken and B. Fady, unpublished). Genetic polymorphisms associated with climate or climate‐related traits (e.g. *Picea sitchensis*, Fig. 3) are generally widespread. Assisted gene flow is unlikely to introduce many adaptive alleles, but it will increase adaptive diversity, frequency of pre‐adapted alleles and frequency of pre‐adapted genotypes compared to local populations.

Increased genetic diversity should provide some buffering in the face of uncertain climates, as many more seedlings are planted than can co‐exist in a mature stand. Natural hybrid populations provide an example of diversity facilitating adaptation across broad climatic conditions. They have higher levels of genetic diversity, on average, than parental species, and this diversity has facilitated adaptation to a wide range of climatic niches. For example, in western North America, *Picea glauca x engelmannii* populations are locally adapted to conditions spanning from boreal to subalpine climates (De La Torre et al. 2014), and *P. glauca x sitchensis* populations are locally adapted to climates ranging from maritime to continental climates (Hamilton and Aitken 2013). Limited introgression occurs among all three species in areas where these major climatic zones meet (Hamilton et al. 2015). In species forming natural hybrid complexes without evidence of outbreeding depression, AGF could be used to plant seedling mixtures with a wide range of hybrid ancestries in source populations, allowing competition and selection to favour those combinations that are well adapted to new climates.

### Avoiding outbreeding depression

AGF is not recommended between long‐diverged evolutionary lineages (e.g. varieties or subspecies) if those lineages and their hybrids have not been well studied, and if populations are expected to naturally regenerate in the future (Aitken and Whitlock 2013). Phylogenetic and phylogeographic studies provide the data necessary to evaluate divergence. Outbreeding depression could result from mixing long‐diverged lineages, although it appears to be less common than previously thought (Whiteley et al. 2015) and is unlikely to occur between populations of wind‐pollinated trees with a continuous distribution. Population simulations suggest natural selection will resolve mild outbreeding depression fairly quickly (Aitken and Whitlock 2013).

### Role of epigenetics

At present, not much is known about the extent of epigenetic effects of maternal environments in forest trees. Research on *Picea abies* is the most comprehensive available. In this species, temperatures of maternal environment during seed maturation can have a profound effect on seedling physiological traits, with seed produced in warmer environments producing delayed fall phenology and decreased cold hardiness, with effect sizes varying genetically among families (Kvaalen and Johnsen 2008). If this effect exists more broadly across tree species, then the clines in Fig. 4A are likely inflated by epigenetic effects of provenance environments where seed was collected. Assisted gene flow can utilize both genetic and epigenetic variation for pre‐adapting forests to climate change. Further research is needed in this area to understand epigenetic effects, but this should not delay implementation of the modest degree of AGF, we recommend here.

### Selectively bred versus natural populations

Optimal climatic distances for AGF may differ between seedlots from selectively bred populations and those from natural populations. Selectively bred reforestation populations will typically have been tested over a range of environments within a breeding zone, and be better characterized than natural populations. Species with selective breeding programs will also usually have comprehensive provenance trials. While in most species, population average growth and cold hardiness are negatively correlated, the effects of selective breeding for increased growth on local adaptation to climate differ among species (Howe et al. 2004). For some species, selection for faster growth will result in delayed budset (e.g. *Larix occidentalis*, Rehfeldt 1995), potentially pre‐adapting material in breeding programs to new climates. For other species, faster growth through selective breeding has been achieved through increased growth rate, not extended growing season duration.

### Insect and disease resistance

We have not reviewed the literature on patterns of insect and disease resistance, but warmer winters, longer growing seasons and changing precipitation regimes are allowing pest and pathogen ranges to expand into areas that were previously too cold (Weed et al. 2013). It is unlikely that AGF from warmer to colder populations will increase susceptibility to currently temperature‐limited insects and diseases compared to planting local, naïve populations that have not recently experienced pressure from these pests. High levels of genetic diversity from AGF may also provide some resilience in the face of changing pressures from both native and exotic pests and pathogens.

### Local adaptation to non‐climatic factors

For some species and environments, local adaptation to non‐climatic factors may also need to be considered (Aitken and Whitlock 2013). Most temperate species cease height growth, set terminal buds, and initiate cold‐hardiness development in response to a critical night length, and long latitudinal transfers northward may disrupt this relationship (Aitken and Whitlock 2013). We recommend AGF be limited to a few degrees of latitude, especially at high latitudes where photoperiod changes more quickly than at lower latitudes. Short latitudinal transfers or longer elevational or longitudinal transfers will not disrupt photoperiodic signals.

While to date there have not been many studies of local adaptation to edaphic factors, AGF of populations or varieties endemic to particular soil types or locally adapted to soil biota may lead to maladaptation, for example with mycorrhizal fungi (Kranabetter et al. 2012; Kranabetter 2014). In *Pseudotsuga menziesii*, a seedling greenhouse common garden was used to test the performance of several provenances with soils from each of those provenances (Pickles et al. 2015). ‘Home’ soils did not result in the highest growth for most populations, and growth was highest, on average, for populations planted in soils from drier locations than their provenances. Survival was higher in fungicide‐treated soil than in non‐treated soil, and showed no local adaptation. However, in non‐fungicide‐treated soil a signature of local adaptation for survival was observed, suggesting complex interactions may exist between fungal and tree populations.

## Summary and recommendations

When Ledig and Kitzmiller (1992) first wrote about the potential for AGF, climate change was far from a certainty. That is no longer the case: the evidence for global climate change is now unequivocal, and it is time for both adaptation and mitigation strategies to be implemented broadly and rapidly. Here, we summarize our recommendations for AGF of forest trees, utilizing the broad knowledge that has accrued on local adaptation over the past several centuries.

1. For species with long‐term field‐based provenance trial data available, AGF strategies can integrate information on provenance productivity and other traits with climate change projections. Assisted gene flow can compensate for climate change and also increase productivity and carbon sequestration by correcting for pre‐global warming adaptational lag, as well as expanding planting areas for exceptionally productive provenances.
2. For species with information on genetic clines from seedling experiments or comprehensive genome scans for adaptation, environmental drivers of local adaptation can be identified and populations adapted to similar climates can be identified and grouped. Assisted gene flow strategies can be designed to match these genetic groups with new climates.
3. For species without studies available on adaptive population divergence along climatic gradients, we suggest designing AGF according to average patterns for sympatric species, or using purely climatic data to match provenances with anticipated site conditions. This will be less risky than ignoring climate change and continuing with reforestation using local seed.
4. Forest managers should consider composite provenancing, mixing seedlings grown from local sources with AGF‐selected non‐local seedlings to increase diversity and resilience, and reduce risk of plantation or restoration failure. Many more trees are planted than can co‐exist to maturity, and competition will thin them over time, retaining the best adapted. The ratio of local to non‐local seedling should depend on species genecological knowledge as well as forest management objectives.
5. To improve forest health and productivity, prioritize sites with high reverse velocities of climate change for AGF to increase forest productivity.
6. To conserve genetic diversity and genotypes adapted to climatic extremes, prioritize AGF of populations near species rear edges to areas expected to persist within the species bioclimate niche.
7. Until seed transfer guidelines or policies are rewritten to include AGF, all seed transfers from colder collection sites to milder planting sites within a seed zone should cease as a stopgap measure.
8. Match populations with middle‐of‐the‐road climate projections for two or so decades in the future. Seedlings need to be able to grow under typical conditions and survive extreme climatic events during establishment. Projections farther into the future would also require choosing among or taking into account more variable and uncertain climate change projections.
9. AGF should be implemented with other silvicultural practices that will increase forest resilience, including mixing species and increasing planting density to allow for greater mortality during establishment.
10. The genetic origin of plantings should be tracked over time to allow for monitoring of health and effects of interactions with biotic and abiotic agents, and to facilitate adaptive management as climates change.

## Supporting information


**Table S1**. Regressions of population mean height vs. provenance mean annual temperature (MAT) and vs. mean summer precipitation (MSP).
**Table S2**. Regressions of population mean timing of spring events vs. provenance mean annual temperature (MAT) and vs. mean summer precipitation (MSP).
**Table S3**. Regressions of population mean timing of fall events vs. provenance mean annual temperature (MAT) and vs. mean summer precipitation (MSP).
**Figure S1.** Adaptive clines in height growth potential along a precipitation gradient are relatively uncommon and inconsistent.
**Figure S2.** Height growth potential vs. provenance mean annual temperature from provenance trials in western North American tree species (Part I: continued in Figure S3).
**Figure S3.** Height growth potential vs. provenance mean annual temperature from provenance trials in western North American tree species (Part II: continued from Figure S2).
**Figure S4.** Height growth potential vs. provenance mean summer precipitation from provenance trials in western North American tree species (Part I: continued in Figure S5).
**Figure S5.** Height growth potential vs. provenance mean summer precipitation from provenance trials in western North American tree species (Part II: continued from Figure S4).
**Figure S6**. Date of spring events (see text) vs. provenance mean annual temperature from provenance trials in western North American tree species.
**Figure S7.** Date of spring events (see text) vs. provenance mean summer precipitation from provenance trials in western North American tree species.
**Figure S8.** Date of fall events (see text) vs. provenance mean annual temperature from provenance trials in western North American tree species. Each box represents an independent provenance trial, and each point represents a single population.
**Figure S9.** Date of fall events (see text) vs. provenance mean summer precipitation from provenance trials in western North American tree species.

## References

[eva12293-bib-0001] Aitken, S. N. , and W. T. Adams 1996 Genetics of fall and winter cold hardiness of coastal Douglas‐fir in Oregon. Canadian Journal of Forest Research 26:1828–1837.

[eva12293-bib-0002] Aitken, S. N. , and M. C. Whitlock 2013 Assisted gene flow to facilitate local adaptation to climate change. Annual Review of Ecology Evolution and Systematics, 44:367–388.

[eva12293-bib-0511] Aitken, S. N. , and J. B. Bemmels 2015 Data from: Time to get moving: assisted migration of forest trees. doi: 10.5061/dryad.rj15h. 10.1111/eva.12293 PMC4780373 27087852

[eva12293-bib-0003] Aitken, S. N. , S. Yeaman , J. A. Holliday , T. L. Wang , and S. Curtis‐McLane 2008 Adaptation, migration or extirpation: climate change outcomes for tree populations. Evolutionary Applications 1:95–111. 25567494 10.1111/j.1752-4571.2007.00013.x PMC3352395

[eva12293-bib-0004] Alberto, F. , S. N. Aitken , R. Alia , S. C. Gonzalez‐Martinez , H. Hanninen , A. Kremer , F. Lefevre et al. 2013 Potential for evolutionary responses to climate change ‐ evidence from tree populations. Global Change Biology 19:1645–1661. 23505261 10.1111/gcb.12181 PMC3664019

[eva12293-bib-0005] Allen, C. D. , A. K. Macalady , H. Chenchouni , D. Bachelet , N. McDowell , M. Vennetier , T. Kitzberger et al. 2010 A global overview of drought and heat‐induced tree mortality reveals emerging climate change risks for forests. Forest Ecology and Management 259:660–684.

[eva12293-bib-0006] Bongarten, B. C. 1978 Genetic and environmental variation in shoot growth and other traits of blue spruce (Picea pungens). PhD thesis, Michigan State University, East Lansing, MI.

[eva12293-bib-0007] Bower, A. D. , and S. N. Aitken 2008 Ecological genetics and seed transfer guidelines for *Pinus albicaulis* (Pinaceae). American Journal of Botany 95:66–76. 21632316 10.3732/ajb.95.1.66

[eva12293-bib-0008] Broadhurst, L. M. , A. Lowe , D. J. Coates , S. A. Cunningham , M. McDonald , P. A. Vesk , and C. Yates 2008 Seed supply for broadscale restoration: maximizing evolutionary potential. Evolutionary Applications 1:587–597. 25567799 10.1111/j.1752-4571.2008.00045.x PMC3352390

[eva12293-bib-0009] Brown, A. G. , and J. C. Doran 1985 Variation in growth and branching characteristics of *Pinus attenuata* . Silvae Genetica 34:100–104.

[eva12293-bib-0010] Cherry, M. L. 1995 Genetic variation in western red cedar (Thuja plicata Donn) seedlings. PhD thesis, University of British Columbia.

[eva12293-bib-0011] Chmura, D. J. , P. D. Anderson , G. T. Howe , C. A. Harrington , J. E. Halofsky , D. L. Peterson , D. C. Shaw et al. 2011 Forest responses to climate change in the northwestern United States: ecophysiological foundations for adaptive management. Forest Ecology and Management 261:1121–1142.

[eva12293-bib-0012] Cieslar, A. 1895 Uber die Erblichkeit des Zuwachsvermogens bei den Waldbaumen. Centralbl. f. das ges. Forstw. 21.

[eva12293-bib-0013] Cieslar, A. 1899 Neues aus dem Gebiet der forstlichen Zuchtwahl. Centralbl. f. das ges. Forstw. 25.

[eva12293-bib-0014] Clausen, J. , D. D. Keck , and W. M. Hiesey 1940 Experimental Studies on the Nature of Species. I. Effects of Varied Environments on Western North American Plants. Carnegie Institution of Washington, Washington, DC.

[eva12293-bib-0015] Clausen, J. , D. D. Keck , and W. M. Hiesey 1948 Experimental Studies on Teh Nature of Species. III. Environmental Responses of Climatic Races of Achillea. Carnegie Institute of Washington, Washington, DC.

[eva12293-bib-0016] Cooke, J. E. K. , M. E. Eriksson , and O. Junttila 2012 The dynamic nature of bud dormancy in trees: environmental control and molecular mechanisms. Plant, Cell and Environment 35:1707–1728. 10.1111/j.1365-3040.2012.02552.x 22670814

[eva12293-bib-0017] Darwin, C. 1859 On the Origin of Species by Means of Natural Selection or the Preservation of Favoured Races in the Struggle for Life. John Murray, London. PMC5184128 30164232

[eva12293-bib-0018] Davis, M. B. , and R. G. Shaw 2001 Range shifts and adaptive responses to quaternary climate change. Science 292:673–679. 11326089 10.1126/science.292.5517.673

[eva12293-bib-0019] Davis, M. B. , R. G. Shaw , and J. R. Etterson 2005 Evolutionary responses to changing climate. Ecology 86:1704–1714.

[eva12293-bib-0020] De La Torre, A. R. , T. L. Wang , B. Jaquish , and S. N. Aitken 2014 Adaptation and exogenous selection in a *Picea glauca x Picea engelmannii* hybrid zone: implications for forest management under climate change. New Phytologist 201:687–699. 24200028 10.1111/nph.12540 PMC4285121

[eva12293-bib-0021] Dow, B. D. , R. A. Cunningham , and J. M. Krupinsky 1998 Fifteen‐year provenance tests of lodgepole pine (*Pinus contorta*) in North Dakota. Western Journal of Applied Forestry 13:5–11.

[eva12293-bib-0022] Duputié, A. , A. Rutschmann , O. Ronce , and I. Chuine 2015 Phenotypic plasticity will not help all species adapt to climate change. Global Change Biology. 21:3062–3073. 25752508 10.1111/gcb.12914

[eva12293-bib-0023] Eckert, A. J. , A. D. Bower , J. L. Wegrzyn , B. Pande , K. D. Jermstad , K. V. Krutovsky , J. B. S. Clair et al. 2009 Asssociation genetics of coastal Douglas‐fir (*Pseudotsuga menziesii* var. *menziesii*, Pinaceae) I. Cold‐hardiness related traits. Genetics 182:1289–1302. 19487566 10.1534/genetics.109.102350 PMC2728866

[eva12293-bib-0025] Engler, A. 1908 Tatsachen, Hypotesen und Irrtumer auf dem Gebiete der Samenprovenienzfrage. Forstwiss. Centralbl. 30.

[eva12293-bib-0026] Enricci, J. A. , N. M. Pasquini , O. A. Picco , and V. Mondino , 2000 Provenance Trials on Pinus ponderosa Douglas ex Lawson in Argentina's Andean Patagonia. Forest Genetic Resources (FAO), vol 1020‐4431, No. 28. Food and Agriculture Organization of the United Nations, Rome, Italy.

[eva12293-bib-0027] Evans, L. M. , G. T. Slavov , E. Rodgers‐Melnick , J. Martin , P. Ranjan , W. Muchero , A. M. Brunner et al. 2014 Population genomics of *Populus trichocarpa* identifies signatures of selection and adaptive trait associations. Nature Genetics 46:1089. 25151358 10.1038/ng.3075

[eva12293-bib-0028] Franks, S. J. , J. J. Weber , and S. N. Aitken 2014 Evolutionary and plastic responses to climate change in terrestrial plant populations. Evolutionary Applications 7:123–139. 24454552 10.1111/eva.12112 PMC3894902

[eva12293-bib-0512] Fuchigami, L. H. , C. J. Weiser , K. D. Kobayashi , R. Timmis , and L. V. Gusta 1982 A degree growth stage (0GS) model and cold acclimation in temperate woody plants In Li P. H., and Sakai A., eds. Plant Cold and Freezing Stress, pp. 93–116. Academic Press, New York, NY.

[eva12293-bib-0029] Gonzalez‐Martinez, S. C. , N. C. Wheeler , E. Ersoz , C. D. Nelson , and D. B. Neale 2007 Association genetics in *Pinus taeda* L I. Wood property traits. Genetics 175:399–409. 17110498 10.1534/genetics.106.061127 PMC1775017

[eva12293-bib-0030] Gonzalez‐Martinez, S. C. , D. Huber , E. Ersoz , J. M. Davis , and D. B. Neale 2008 Association genetics in *Pinus taeda* L II. Carbon isotope discrimination. Heredity 101:19–26. 18478029 10.1038/hdy.2008.21

[eva12293-bib-0031] Gray, L. K. , and A. Hamann 2013 Tracking suitable habitat for tree populations under climate change in western North America. Climatic Change 117:289–303.

[eva12293-bib-0032] Günther, T. , and G. Coop 2013 Robust identification of local adaptation from allele frequencies. Genetics 195:205–220. 23821598 10.1534/genetics.113.152462 PMC3761302

[eva12293-bib-0034] Hamann, A. , D. R. Roberts , Q. E. Barber , C. Carroll , and S. E. Nielsen 2014 Velocity of climate change algorithms for guiding conservation and management. Global Change Biology 21:997–1004. 25310933 10.1111/gcb.12736

[eva12293-bib-0035] Hamilton, J. A. , and S. N. Aitken 2013 Genetic and morphological structure of a spruce hybrid (*Picea sitchensis x P. glauca*) zone along a climatic gradient. American Journal of Botany 100:1651–1662. 23935108 10.3732/ajb.1200654

[eva12293-bib-0036] Hamilton, J. A. , A. R. De La Torre , and S. N. Aitken 2015 Fine‐scale environmental variation contributes to introgression in a three‐species spruce hybrid complex. Tree Genetics and Genomes 11:1–14.

[eva12293-bib-0037] Hampe, A. , and R. J. Petit 2005 Conserving biodiversity under climate change: the rear edge matters. Ecology Letters 8:461–467. 21352449 10.1111/j.1461-0248.2005.00739.x

[eva12293-bib-0038] Hannerz, M. , S. N. Aitken , J. N. King , and S. Budge 1999 Effects of genetic selection for growth on frost hardiness in western hemlock. Canadian Journal of Forest Research 29:509–516.

[eva12293-bib-0039] Hansen, O. K. , U. B. Nielsen , Ø. M. Edvardsen , B. Skúlason , and J.‐O. Skage 2004 Nordic provenance trials with *Abies lasiocarpa* and *Abies lasiocarpa* var. *arizonica*: three‐year results. Scandinavian Journal of Forest Research 19:112–126.

[eva12293-bib-0040] Harrington, C. A. , and P. J. Gould 2015 Tradeoffs between chilling and forcing in satisfying dormancy requirements for Pacific Northwest tree species. Frontiers in Plant Science 6:1–12. 25784922 10.3389/fpls.2015.00120 PMC4347443

[eva12293-bib-0041] Hijmans, R. J. , S. E. Cameron , J. L. Parra , P. G. Jones , and A. Jarvis 2005 Very high resolution interpolated climate surfaces for global land areas. International Journal of Climatology 25:1965–1978.

[eva12293-bib-0042] Holliday, J. A. , K. Ritland , and S. N. Aitken 2010a Widespread, ecologically relevant genetic markers developed from association mapping of climate‐related traits in Sitka spruce (*Picea sitchensis*). New Phytologist 188:501–514. 20663060 10.1111/j.1469-8137.2010.03380.x

[eva12293-bib-0043] Holliday, J. A. , M. Yuen , K. Ritland , and S. N. Aitken 2010b Postglacial history of a widespread conifer produces inverse clines in selective neutrality tests. Molecular Ecology 19:3857–3864. 20738783 10.1111/j.1365-294X.2010.04767.x

[eva12293-bib-0044] Holliday, J. A. , H. Suren , and S. N. Aitken 2012 Divergent selection and heterogeneous migration rates across the range of Sitka spruce (*Picea sitchensis*). Proceedings of the Royal Society B‐Biological Sciences 279:1675–1683. 10.1098/rspb.2011.1805 PMC3297444 22113032

[eva12293-bib-0045] Howe, G. T. , S. N. Aitken , D. B. Neale , K. D. Jermstad , N. C. Wheeler , and T. H. H. Chen 2004 From genotype to phenotype: unraveling the complexities of cold adaptation in forest trees. Canadian Journal of Botany 81:1247–1266.

[eva12293-bib-0046] Huebert, C. A. 2009 The ecological and conservation genetics of Garry oak (Quercus garryana Dougl. ex Hook). M.Sc. thesis, University of British Columbia.

[eva12293-bib-0047] Illingworth, K. 1978 Study of lodgepole pine genotype‐environment interaction in B.C. In: International Union of Forestry Research Organizations (IUFRO) Joint Meeting of Working Parties: Douglas‐fir Provenances, Lodgepole Pine provenances, Sitka Spruce Provenances and Abies Provenances, Vancouver, British Columbia, Canada. pp 151‐158.

[eva12293-bib-0048] Jermstad, K. D. , D. L. Bassoni , K. S. Jech , N. C. Wheeler , and D. B. Neale 2001a Mapping of quantitative trait loci controlling adaptive traits in coastal Douglas‐fir. I. Timing of vegetative bud flush. Theoretical and Applied Genetics 102:1142–1151. 10.1093/genetics/165.3.1489 PMC1462859 14668397

[eva12293-bib-0049] Jermstad, K. D. , D. L. Bassoni , N. C. Wheeler , T. S. Anekonda , S. N. Aitken , W. T. Adams , and D. B. Neale 2001b Mapping of quantitative trait loci controlling adaptive traits in coastal Douglas‐fir. II. Spring and fall cold‐hardiness. Theoretical and Applied Genetics 102:1152–1158.

[eva12293-bib-0050] Keir, K. R. , J. B. Bemmels , and S. N. Aitken 2011 Low genetic diversity, moderate local adaptation, and phylogeographic insights in *Cornus nuttallii* (Cornaceae). American Journal of Botany 98:1327–1336. 21821593 10.3732/ajb.1000466

[eva12293-bib-0051] Kirkpatrick, M. , and N. H. Barton 1997 Evolution of a species' range. American Naturalist 150:1–23. 10.1086/286054 18811273

[eva12293-bib-0052] Klinka, K. , J. Worrall , L. Skoda , and P. Varga 2000 The Distribution and Synopsis of Ecological and Silvical Characteristics of Tree Species of British Columbia's Forests. Canadian Cartographics Ltd, Coquitlam, BC, Canada.

[eva12293-bib-0053] König, A. 1995 Geographic variation of *Abies grandis*‐provenances grown in northwestern Germany. Silvae Genetica 44:248–255.

[eva12293-bib-0054] Kozlowski, T. T. , and S. G. Pallardy 2002 Acclimation and adaptive responses of woody plants to environmental stresses. The Botanical Review 68:270–334.

[eva12293-bib-0055] Kranabetter, J. M. 2014 Ectomycorrhizal fungi and the nitrogen economy of conifers‐implications for genecology and climate change mitigation. Botany‐Botanique 92:417–423.

[eva12293-bib-0056] Kranabetter, J. M. , M. U. Stoehr , and G. A. O'Neill 2012 Divergence in ectomycorrhizal communities with foreign Douglas‐fir populations and implications for assisted migration. Ecological Applications 22:550–560. 22611853 10.1890/11-1514.1

[eva12293-bib-0057] Kremer, A. , O. Ronce , J. J. Robledo‐Arnuncio , F. Guillaume , G. Bohrer , R. Nathan , J. R. Bridle et al. 2012 Long‐distance gene flow and adaptation of forest trees to rapid climate change. Ecology Letters 15:378–392. 22372546 10.1111/j.1461-0248.2012.01746.x PMC3490371

[eva12293-bib-0058] Krutzsch, P. 1992 IUFRO's role in coniferous tree improvement: Norway spruce (*Picea abies* (L.) Karst.). Silvae Genetica 41:143–150.

[eva12293-bib-0060] Kuser, J. E. , and K. K. Ching 1981 Provenance variation in seed weight, cotyledon number, and growth rate of western hemlock seedlings. Canadian Journal of Forest Research 11:662–670.

[eva12293-bib-0061] Kvaalen, H. , and O. Johnsen 2008 Timing of bud set in *Picea abies* is regulated by a memory of temperature during zygotic and somatic embryogenesis. New Phytologist 177:49–59. 17924949 10.1111/j.1469-8137.2007.02222.x

[eva12293-bib-0062] Langlet, O. 1971 Two hundred years of genecology. Taxon 20:653–722.

[eva12293-bib-0063] Latta, R. G. 2003 Gene flow, adaptive population divergence and comparative population structure across loci. New Phytologist 161:51–58.

[eva12293-bib-0065] Le Corre, V. , and A. Kremer 2012 The genetic differentiation at quantitative trait loci under local adaptation. Molecular Ecology 21:1548–1566. 22332667 10.1111/j.1365-294X.2012.05479.x

[eva12293-bib-0066] Ledig, F. T. , and J. H. Kitzmiller 1992 Genetic strategies for reforestation in the face of global climate change. Forest Ecology and Management 50:153–169.

[eva12293-bib-0067] Leimu, R. , and M. Fischer . 2008 A meta‐analysis of local adaptation in plants. PLoS ONE 3:e4010. 19104660 10.1371/journal.pone.0004010 PMC2602971

[eva12293-bib-0068] Little, E. L. J. . 1971 Atlas of United States Trees, Volume 1, Conifers and Important Hardwoods. U.S. Department of Agriculture Forest Service Miscellaneous Publication 1146, Washington, DC.

[eva12293-bib-0069] Little, E. L. J. . 1976 Atlas of United States Trees, Volume 3, Minor Western Hardwoods. U.S. Department of Agriculture Forest Service Miscellaneous Publication 1314, Washington, DC.

[eva12293-bib-0070] Loarie, S. R. , P. B. Duffy , H. Hamilton , G. P. Asner , C. B. Field , and D. D. Ackerly 2009 The velocity of climate change. Nature 462:1052–1055. 20033047 10.1038/nature08649

[eva12293-bib-0071] Lobo, N. L. 2011 Clinal variation at putatively adaptive polymorphisms in mature populations of Sitka spruce (Picea sitchensis (Bong.) Carr.). MSc Thesis, University of British Columbia.

[eva12293-bib-0072] Lotterhos, K. E. , and M. C. Whitlock 2015 The relative power of genome scans to detect local adaptation depends on sampling design and statistical method. Molecular Ecology 24:1031–1046. 25648189 10.1111/mec.13100

[eva12293-bib-0513] Mátyás, C. . 1994 Modeling climate‐change effects with provenance test data. Tree Physiology 14:797–804. 14967649 10.1093/treephys/14.7-8-9.797

[eva12293-bib-0073] Matthew, P. 1831 On naval timber and arboriculture; with critical notes on authors who have recently treated the subject of planting. Edinburgh and London (available from Google Books), Black.

[eva12293-bib-0075] McDowell, N. , W. T. Pockman , C. D. Allen , D. D. Breshears , N. Cobb , T. Kolb , J. Plaut et al. 2008 Mechanisms of plant survival and mortality during drought: why do some plants survive while others succumb to drought? New Phytologist 178:719–739. 18422905 10.1111/j.1469-8137.2008.02436.x

[eva12293-bib-0076] McKown, A. D. , R. D. Guy , J. Klápště , A. Geraldes , M. Friedmann , Q. C. B. Cronk , Y. A. El‐Kassaby et al. 2013 Geographical and environmental gradients shape phenotypic trait variation and genetic structure in *Populus trichocarpa* . New Phytologist 201:1263–1276. 24491114 10.1111/nph.12601

[eva12293-bib-0077] Mimura, M. , and S. N. Aitken 2007a Adaptive gradients and isolation‐by‐distance with postglacial migration in *Picea sitchensis* . Heredity 99:224–232. 17487214 10.1038/sj.hdy.6800987

[eva12293-bib-0078] Mimura, M. , and S. N. Aitken 2007b Increased selfing and decreased effective pollen donor number in peripheral relative to central populations in *Picea sitchensis* (*Pinaceae*). American Journal of Botany 94:991–998. 21636468 10.3732/ajb.94.6.991

[eva12293-bib-0079] Pickles, B. J. , B. D. Twieg , G. A. O'Neill , W. W. Mohn , and S. W. Simard 2015 Local adaptation in migrated interior Douglas‐fir seedlings is mediated by ectomycorrhizas and other soil factors. New Phytologist 207:858–871. 25757098 10.1111/nph.13360

[eva12293-bib-0080] Porter, R. B. , T. Lacourse , B. J. Hawkins , and A. Yanchuk . 2013 Adaptive variation in growth, phenology, cold tolerance and nitrogen fixation of red alder (*Alnus rubra* Bong.). Forest Ecology and Management 291:357–366.

[eva12293-bib-0081] Read, R. A. 1980 Genetic variation in seedling progeny of Ponderosa pine provenances. Forest Science Monographs 23:1–60.

[eva12293-bib-0082] Rehfeldt, G. E. 1994 Adaptation of *Picea engelmannii* populations to the heterogeneous environments of the Intermountain West. Canadian Journal of Botany 72:1197–1208.

[eva12293-bib-0083] Rehfeldt, G. E. 1995 Genetic‐variation, climate models and the ecological genetics of *Larix occidentalis* . Forest Ecology and Management 78:21–37.

[eva12293-bib-0084] Rehfeldt, G. E. , R. J. Hoff , and R. J. Steinhoff 1984 Geographic patterns of genetic variation in *Pinus monticola* . Botanical Gazette 145:229–239.

[eva12293-bib-0085] Rehfeldt, G. E. , C. C. Ying , D. L. Spittlehouse , and D. A. Hamilton 1999 Genetic responses to climate in *Pinus contorta*: Niche breadth, climate change, and reforestation. Ecological Monographs 69:375–407.

[eva12293-bib-0086] Rohde, A. , and R. P. Bhalerao 2007 Plant dormancy in the perennial context. Trends in Plant Science 12:217–223. 17416545 10.1016/j.tplants.2007.03.012

[eva12293-bib-0087] Rohde, A. , V. Storme , V. Jorge , M. Gaudet , N. Vitacolonna , F. Fabbrini , T. Ruccink et al. 2011 Bud set in poplar – genetic dissection of a complex trait in natural and hybrid populations. New Phytologist 189:106–121. 21039557 10.1111/j.1469-8137.2010.03469.x

[eva12293-bib-0088] Sala, A. , D. R. Woodruff , and F. C. Meinzer 2012 Carbon dynamics in trees: feast or famine? Tree Physiology 32:764–775. 22302370 10.1093/treephys/tpr143

[eva12293-bib-0089] Savolainen, O. , T. Pyhajarvi , and T. Knurr . 2007 Gene flow and local adaptation in trees. Annual Review of Ecology Evolution and Systematics 38:595–619.

[eva12293-bib-0090] Savolainen, O. , M. Lascoux , and J. Merilä 2013 Ecological genomics of local adaptation. Nature Reviews Genetics 14:807–820. 10.1038/nrg3522 24136507

[eva12293-bib-0091] Soolanayakanahally, R. Y. , R. D. Guy , S. N. Silim , and M. H. Song 2013 Timing of photoperiodic competency causes phenological mismatch in balsam poplar (*Populus balsamifera* L.). Plant, Cell and Environment 36:116–127. 10.1111/j.1365-3040.2012.02560.x 22702736

[eva12293-bib-0092] Soularue, J. P. , and A. Kremer 2014 Evolutionary responses of tree phenology to the combined effects of assortative mating, gene flow and divergent selection. Heredity 113:485–494. 24924591 10.1038/hdy.2014.51 PMC4815586

[eva12293-bib-0093] Spitze, K. 1993 Population structure in *Daphnia obtusa* ‐ quantitative genetic and allozymic variation. Genetics 135:367–374. 8244001 10.1093/genetics/135.2.367 PMC1205642

[eva12293-bib-0094] St Clair, J. B. , and G. T. Howe 2007 Genetic maladaptation of coastal Douglas‐fir seedlings to future climates. Global Change Biology 13:1441–1454.

[eva12293-bib-0095] St Clair, J. B. , N. L. Mandel , and K. W. Vance‐Boland 2005 Genecology of Douglas‐fir in western Oregon and Washington. Annals of Botany 96:1199–1214. 16246849 10.1093/aob/mci278 PMC4247077

[eva12293-bib-0096] Sweet, G. B. 1965 Provenance differences in Pacific coast Douglas‐fir. Silvae Genetica 14:46–56.

[eva12293-bib-0097] Thompson, R. S. , K. H. Anderson , and P. J. Bartlein . 2000 Atlas of Relations Between Climatic Parameters and Distributions of Important Trees and Shrubs in North America. U.S. Geological Survey Professional Paper 1650‐C, Reston, VA, 386 pp.

[eva12293-bib-0098] Turesson, G. 1923 The scope and import of genecology. Hereditas 4:171–176.

[eva12293-bib-0099] Vilmorin, P. P. A. D. 1862 Expose historique et descriptif de l'Ecole forestiere des Barres pres de Nogent‐sur‐Vernisson (Loiret). Mem. Soc. Imp. Centr. d'Agric, France, Paris.

[eva12293-bib-0100] Vitasse, Y. , C. C. Bresson , A. Kremer , R. Michalet , and S. Delzon 2010 Quantifying phenological plasticity to temperature in two temperate tree species. Functional Ecology 24:1211–1218.

[eva12293-bib-0101] Wagenheim, F. A. J. V. 1787 Beytrag zur teutschen holzgerechten Forstwissenschaft, die Anpflanzung Nordamericanischer Holzarten, mit Anwendung auf teutsche Forste, Gottingen.

[eva12293-bib-0102] Wang, T. , A. Hamann , A. Yanchuk , G. A. O'Neill , and S. N. Aitken 2006 Use of response functions in selecting lodgepole pine populations for future climate. Global Change Biology 12:2404–2416.

[eva12293-bib-0103] Wang, T. L. , G. A. O'Neill , and S. N. Aitken 2010 Integrating environmental and genetic effects to predict responses of tree populations to climate. Ecological Applications 20:153–163. 20349837 10.1890/08-2257.1

[eva12293-bib-0104] Wang, T. , A. Hamann , D. L. Spittlehouse , and T. Q. Murdock 2012 ClimateWNA—High‐resolution spatial climate data for western North America. Journal of Applied Meteorology and Climatology 51:16–29.

[eva12293-bib-0514] Weed, A. S. , M. P. Ayres , and J. A. Hicke . 2013 Consequences of climate change for biotic disturbances in North American Forests. Ecological Mongraphs 83:441–470.

[eva12293-bib-0105] White, T. L. 1987 Drought tolerance of southwestern Oregon Douglas‐fir. Forest Science 33:283–293.

[eva12293-bib-0106] White, T. L. , and K. K. Ching 1985 Provenance study of Douglas‐fir in the Pacific Northwest region. IV. Field performance at age 25 years. Silvae Genetica 34:84–90.

[eva12293-bib-0108] Whiteley, A. R. , S. W. Fitzpatrick , and W. C. Funk 2015 Genetic rescue to the rescue. Trends in Ecology & Evolution 30:42–49. 25435267 10.1016/j.tree.2014.10.009

[eva12293-bib-0109] Whitlock, M. C. 2008 Evolutionary inference from *Q* _ST_ . Molecular Ecology 17:1885–1896. 18363667 10.1111/j.1365-294X.2008.03712.x

[eva12293-bib-0110] Whitlock, M. C. , and F. Guillaume 2009 Testing for spatially divergent selection: comparing *Q* _ST_ to *F* _ST_ . Genetics 183:1055–1063. 19687138 10.1534/genetics.108.099812 PMC2778959

[eva12293-bib-0111] Wright, J. W. , W. A. Lemmien , and J. N. Bright 1971 Genetic variation in Southern Rocky Mountain white fir. Silvae Genetica 20:148–150.

[eva12293-bib-0112] Xie, C. Y. , and C. C. Ying 1993 Geographic variation of grand fir (*Abies grandis*) in the Pacific coast region: 10‐year results from a provenance trial. Canadian Journal of Forest Research 23:1065–1072.

[eva12293-bib-0113] Xie, C. Y. , and C. C. Ying 1994 Adaptedness of noble fir (*Abies procera* Rehd.) beyond its northern limit. Forest Science 40:412–428.

[eva12293-bib-0114] Yeaman, S. , and A. Jarvis 2006 Regional heterogeneity and gene flow maintain variance in a quantitative trait within populations of lodgepole pine. Proceedings of the Royal Society B‐Biological Sciences 273:1587–1593. 10.1098/rspb.2006.3498 PMC1634926 16769628

[eva12293-bib-0115] Ying, C. C. 1997 Effects of site, provenance, and provenance and site interaction in Sitka spruce in coastal British Columbia. Forest Genetics 4:99–112.

