# Supplementary material for: Time to get moving: assisted gene flow of forest trees
Source: Evol Appl. 2015 Aug 24;9(1):271–90. doi: 10.1111/eva.12293 (PMC4780373; doi:10.1111/eva.12293)
Supplement: Supplementary file 1 — Table S1. Regressions of population mean height vs. provenance mean annual temperature (MAT) and vs. mean summer precipitation (MSP). Table S2. Regressions of population mean timing of spring events vs. provenance mean annual temperature (MAT) and vs. mean summer precipitation (MSP). Table S3. Regressions of population mean timing of fall events vs. provenance mean annual temperature (MAT) and vs. mean summer precipitation (MSP). Figure S1. Adaptive clines in height growth potential along a precipitation gradient are relatively uncommon and inconsistent. Figure S2. Height growth potential vs. provenance mean annual temperature from provenance trials in western North American tree species (Part I: continued in Figure S3). Figure S3. Height growth potential vs. provenance mean annual temperature from provenance trials in western North American tree species (Part II: continued from Figure S2). Figure S4. Height growth potential vs. provenance mean summer precipitation from provenance trials in western North American tree species (Part I: continued in Figure S5). Figure S5. Height growth potential vs. provenance mean summer precipitation from provenance trials in western North American tree species (Part II: continued from Figure S4). Figure S6. Date of spring events (see text) vs. provenance mean annual temperature from provenance trials in western North American tree species. Figure S7. Date of spring events (see text) vs. provenance mean summer precipitation from provenance trials in western North American tree species. Figure S8. Date of fall events (see text) vs. provenance mean annual temperature from provenance trials in western North American tree species. Each box represents an independent provenance trial, and each point represents a single population. Figure S9. Date of fall events (see text) vs. provenance mean summer precipitation from provenance trials in western North American tree species. [file EVA-9-271-s001.pdf]

Supplementary Table 1.

Regressions of population mean height vs. provenance mean annual temperature (MAT) and vs. mean summer precipitation (MSP).

Variable definitions: m = standardized regression slope; n_pops = number of populations; n_families = number of open-pollinated families per population, if known; n_individs = number of individuals per population, if known; age = age of trees when height was measured; study area = geographic origin of provenances.

	**MAT**			**MSP**								
**Species**	**m (%·°C^{-1})**	**R^2**	**p**	**m (%·(100mm)$^{-1}$)**	**R^2**	**p**	**n_pops**	**n_families**	**n_individs**	**Age**	**Study area***	**Reference**
Abies concolor	**4.7**	**0.28**	**0.0422**	9.3	0.15	0.1567	15	3 to 100	–	8	Southern Rockies	Wright et al. 1971
Abies grandis	**7.5**	**0.60**	**<0.0001**	4.0	0.02	0.4845	22	–	–	15	BC & Central Coasts	König 1995
Abies grandis	-0.1	0.00	0.9743	2.5	0.04	0.3762	21	15 to 25	c. 270	10	Central Coast	Xie and Ying 1993
Abies lasiocarpa	**1.7**	**0.32**	**0.0295**	-3.2	0.19	0.1046	15	–	c. 155†	3	Alaska & Central Coasts; Boreal; Northern, Central & Southern Rockies	Hansen et al. 2004
Abies procera	-2.6	0.14	0.1509	2.1	0.02	0.5591	16	–	43 to 56	10	Central Coast	Xie and Ying 1994
Cornus nuttallii	**3.9**	**0.51**	**0.0315**	-5.4	0.25	0.1718	9	3 to 20	12 to 169	1	BC, Central & Southern Coasts	Keir et al. 2011
Picea engelmannii	**3.5**	**0.19**	**<0.0001**	-3.3	0.02	0.1855	103	bulk	27†	2	Central & Southern Rockies; Intermountain	Rehfeldt 1994
Picea pungens	**3.6**	**0.32**	**<0.0001**	**12.8**	**0.28**	**0.0007**	38	c. 7	–	7	Central & Southern Rockies	Bongarten 1978
Picea sitchensis	**13.4**	**0.85**	**<0.0001**	**-8.6**	**0.48**	**0.0022**	17	10 to 13; bulk	30 to 124	3	Alaska, BC & Central Coasts	Mimura and Aitken 2007
Picea sitchensis	**6.9**	**0.89**	**<0.0001**	-2.6	0.24	0.1507	10	–	79 to 81	20	Alaska, BC & Central Coasts	Ying 1997
Pinus albicaulis	2.7	0.02	0.5461	3.9	0.02	0.4576	25	3 to 17	15 to 89	3	BC & Central Coasts; Sierra Nevada; Central Rockies; Intermountain	Bower and Aitken 2008
Pinus attenuata	**7.4**	**0.30**	**0.0007**	**-17.2**	**0.37**	**0.0002**	32	5 to 6; –	9 to 27	8	Central & Southern Coasts; Sierra Nevada	Brown and Doran 1985
Pinus contorta	1.7	0.06	0.2592	-1.9	0.01	0.6668	23	–	–	15	Northern, Central & Southern Rockies; Western Great Plains	Dow et al. 1998
Pinus contorta	**2.6**	**0.52**	**<0.0001**	0.0	0.00	0.9767	54	–	–	20	Alaska & BC Coasts; Boreal; Northern Rockies; Intermountain	Illingworth 1978
Pinus monticola	0.6	0.00	0.6415	**10.3**	**0.28**	**<0.0001**	60	≥ 5	30†	3	BC & Central Coasts; Sierra Nevada; Northern & Central Rockies; Intermountain	Rehfeldt et al. 1984
Pinus ponderosa	1.0	0.06	0.3377	2.2	0.05	0.3805	17	–	36†	16	Central Coast; Northern Rockies; Intermountain	Enricci et al. 2000
Pinus ponderosa	**5.1**	**0.27**	**<0.0001**	**11.3**	**0.11**	**0.0049**	72	7 to 17	c. 100	3	Southern Rockies; Western Great Plains	Read 1980
Populus trichocarpa	**8.7**	**0.49**	**<0.0001**	0.8	0.00	0.6460	97	3 to 7‡	3 to 7‡	4	Alaska, BC & Central Coasts; Boreal; Northern Rockies	McKown et al. 2013
Pseudotsuga menziesii	5.1	0.13	0.0962	-12.7	0.15	0.0700	23	10	c. 78	2	Central & Southern Coasts; Sierra Nevada	Sweet 1965
Pseudotsuga menziesii	0.5	0.09	0.3231	1.0	0.09	0.3116	13	14 to 89	25†	25	BC & Central Coasts	White and Ching 1985
Quercus garryana	1.8	0.04	0.5019	-17.7	0.30	0.0551	13	9 to 13	108 to 156	2	BC & Central Coasts; Sierra Nevada	Huebert 2009
Thuja plicata	**1.9**	**0.30**	**0.0148**	-1.0	0.05	0.3533	19	5; bulk	90 to 450†	3	BC Coast; Northern & Central Rockies	Cherry 1995
Tsuga heterophylla	**2.2**	**0.21**	**0.0354**	**-2.5**	**0.21**	**0.0385**	21	2; bulk	96 to 192	1	Alaska, BC & Central Coasts; Northern & Central Rockies	Kuser and Ching 1981

* Study area definitions:

Alaska Coast – southern Alaska and Alaska panhandle, including adjacent coastal mountains in British Columbia and the Yukon
BC Coast – coastal British Columbia
Central Coast – coastal Washington, Oregon, and northern California; Cascade Mountains
South Coast, coastal California south of San Francisco
Sierra Nevada – Sierra Nevada; inland California
Boreal – interior northern Alberta, British Columbia and the Yukon
Northern Rockies – Canadian Rockies; interior southern British Columbia
Central Rockies – Rocky Mountains in Idaho, Montana, and Wyoming
Southern Rockies – Rocky Mountains in Arizona, Colorado, New Mexico, and Utah
Intermountain – Great Basin; interior Oregon and Washington
Western Great Plains – foothills east of the Rocky Mountains, in Canada and the United States

† Estimate before accounting for mortality.
‡ Number of distinct clonal genotypes.

Supplementary Table 2.

Regressions of population mean timing of spring events vs. provenance mean annual temperature (MAT) and vs. mean summer precipitation (MSP).

Variable definitions: m = standardized regression slope; n_pops = number of populations; n_families = number of open-pollinated families per population, if known; n_individs = number of individuals per population, if known; age = age of trees when height was measured; study area = geographic origin of provenances.

	MAT			MSP									
**Species**	**m ($d \cdot °C^{-1}$)**	**R^2**	**p**	**m ($d \cdot (100mm)^{-1}$)**	**R^2**	**p**	**n_pops**	**n_families**	**n_individs**	**Age**	**Event**	**Study area***	**Reference**
Alnus rubra	**0.7**	**0.40**	**0.0263**	**-0.5**	**0.58**	**0.0038**	12	–	28†	3	bud flush	Alaska, BC & Central Coasts; Central Rockies	Cannell et al. 1987
Alnus rubra	**1.1**	**0.29**	**<0.0001**	-0.1	0.01	0.4549	55	–	100	2	bud flush	BC Coast	Hamann et al. 1998
Picea engelmannii	0.1	0.01	0.3160	0.3	0.03	0.1045	103	bulk	27†	2	bud flush	Central & Southern Rockies; Intermountain	Rehfeldt 1994
Picea pungens	-0.1	0.01	0.6441	**-1.8**	**0.19**	**0.0091**	36	c. 7	–	7	bud flush	Central and Southern Rockies	Bongarten 1978
Picea sitchensis	0.3	0.08	0.2589	-0.2	0.04	0.4402	17	10 to 13; bulk	30 to 124	2	bud flush	Alaska, BC & Central Coasts	Mimura and Aitken 2007
Pinus albicaulis	**2.7**	**0.46**	**0.0002**	**-2.1**	**0.18**	**0.0331**	25	3 to 17	15 to 89	3	bud flush	BC & Central Coasts; Sierra Nevada; Central Rockies; Intermountain	Bower and Aitken 2008
Populus trichocarpa	-0.6	0.02	0.1396	**1.8**	**0.10**	**0.0016**	97	3 to 7‡	3 to 7‡	3	bud flush	Alaska, BC & Central Coasts; Boreal; Northern Rockies	McKown et al. 2013
Pseudotsuga menziesii	**-5.1**	**0.54**	**0.0027**	-0.5	0.00	0.8808	14	–	–	–	bud flush	Northern Rockies	Lavadinović et al. 2013
Pseudotsuga menziesii	-1.1	0.10	0.1379	2.9	0.14	0.0843	23	10	45	2	bud flush	Central & Southern Coasts; Sierra Nevada	Sweet 1965
Pseudotsuga menziesii	**-0.9**	**0.26**	**0.0425**	1.2	0.18	0.1061	16	–	98†	7	bud flush	BC & Central Coasts	White et al. 1979
Tsuga heterophylla	**4.6**	**0.82**	**<0.0001**	0.3	0.00	0.8515	14	3; bulk	40 to 120†	2	bud flush	Alaska, BC & Central Coasts; Northern & Central Rockies	Kuser 1980

* Study area definitions:

Alaska Coast – southern Alaska and Alaska panhandle, including adjacent coastal mountains in British Columbia and the Yukon

BC Coast – coastal British Columbia

Central Coast – coastal Washington, Oregon, and northern California; Cascade Mountains

South Coast, coastal California south of San Francisco

Sierra Nevada – Sierra Nevada; inland California

Boreal – interior northern Alberta, British Columbia and the Yukon

Northern Rockies – Canadian Rockies; interior southern British Columbia

Central Rockies – Rocky Mountains in Idaho, Montana, and Wyoming

Southern Rockies – Rocky Mountains in Arizona, Colorado, New Mexico, and Utah

Intermountain – Great Basin; interior Oregon and Washington

Western Great Plains – foothills east of the Rocky Mountains, in Canada and the United States

† Estimate before accounting for mortality.

‡ Number of distinct clonal genotypes.

Supplementary Table 3.
Regressions of population mean timing of fall events vs. provenance mean annual temperature (MAT) and vs. mean summer precipitation (MSP).
Variable definitions: m = standardized regression slope; n_pops = number of populations; n_families = number of open-pollinated families per population, if known; n_individs = number of individuals per population, if known;
age = age of trees when height was measured; study area = geographic origin of provenances.

	MAT			MSP									
Species	m (d·°C^{-1})	R^2	p	m (d·(100mm)$^{-1}$)	R^2	p	n_pops	n_families	n_individs	Age	Event	Study area*	Reference
Alnus rubra	**4.8**	**0.69**	**0.0008**	**-2.7**	**0.66**	**0.0012**	12	–	44†	2	bud set	Alaska, BC & Central Coasts; Central Rockies	Cannell et al. 1987
Alnus rubra	**2.7**	**0.45**	**<0.0001**	**-0.7**	**0.13**	**0.0077**	55	–	100	1	leaf abscission	BC Coast	Hamann et al. 1998
Picea engelmannii	**0.9**	**0.25**	**<0.0001**	-0.1	0.00	0.8297	102	bulk	27†	2	growth cessation	Central & Southern Rockies; Intermountain	Rehfeldt 1994
Picea sitchensis	**11.4**	**0.87**	**<0.0001**	**-7.1**	**0.47**	**0.0024**	17	10 to 13; bulk	30 to 124	2	bud set	Alaska, BC & Central Coasts	Mimura and Aitken 2007
Populus trichocarpa	**10.6**	**0.62**	**<0.0001**	-0.5	0.00	0.7806	97	3 to 7‡	3 to 7‡	2	bud set	Alaska, BC & Central Coasts; Boreal; Northern Rockies	McKown et al. 2013
Tsuga heterophylla	**7.5**	**0.90**	**<0.0001**	**-4.9**	**0.29**	**0.0179**	19	3; bulk	40 to 120†	1	bud set	Alaska, BC & Central Coasts; Northern & Central Rockies	Kuser 1980

* Study area definitions:
Alaska Coast – southern Alaska and Alaska panhandle, including adjacent coastal mountains in British Columbia and the Yukon
BC Coast – coastal British Columbia
Central Coast – coastal Washington, Oregon, and northern California; Cascade Mountains
South Coast, coastal California south of San Francisco
Sierra Nevada – Sierra Nevada; inland California
Boreal – interior northern Alberta, British Columbia and the Yukon
Northern Rockies – Canadian Rockies; interior southern British Columbia
Central Rockies – Rocky Mountains in Idaho, Montana, and Wyoming
Southern Rockies – Rocky Mountains in Arizona, Colorado, New Mexico, and Utah
Intermountain – Great Basin; interior Oregon and Washington
Western Great Plains – foothills east of the Rocky Mountains, in Canada and the United States
† Estimate before accounting for mortality.
‡ Number of distinct clonal genotypes.

Supplementary Figure 1. Adaptive clines in height growth potential along a precipitation gradient are relatively uncommon and inconsistent. (A) Regression slopes of standardized height growth potential vs. provenance mean summer precipitation, for populations grown in a common test environment. Each line represents an independent provenance trial, and line type corresponds to the species and reference indicated in the legend. Only statistically significant regression slopes are shown ($p < 0.05$). (B) The climatic scope of provenance-trial datasets compared to the species realized niche. For each species, the bottom line represents the core climatic niche (solid black line) and full climatic niche (dashed extension) in mean July precipitation (MJP) (see text; Thompson et al. 2000). The upper lines represent the range of provenance MJP values among populations within each provenance trial for that species. Open circles represent the MJP at the test site(s) for each provenance trial. Provenance trials missing an open circle were grown in a controlled environment. Note that the x-axis scale in (B) does not cover the full range of data for some species; this scaling was applied to increase visibility for the majority of species.

Supplementary Figure 2. Height growth potential vs. provenance mean annual temperature from provenance trials in western North American tree species (Part I: continued in Supp. Fig. 3). Each box represents an independent provenance trial, and each point represents a single population. Slopes are depicted for statistically significant regressions ($p < 0.05$). Species abbreviations consist of the first four letters of the genus, plus the last two letters of the specific epithet (see Fig. 4 for full names). Scale is not comparable among boxes; axes were rescaled independently for maximum visibility of the spread of data.

Supplementary Figure 3. Height growth potential vs. provenance mean annual temperature from provenance trials in western North American tree species (Part II: continued from Supp. Fig. 2). Each box represents an independent provenance trial, and each point represents a single population. Slopes are depicted for statistically significant regressions ($p < 0.05$). Species abbreviations consist of the first four letters of the genus, plus the last two letters of the specific epithet (see Fig. 4 for full names). Scale is not comparable among boxes; axes were rescaled independently for maximum visibility of the spread of data.

Supplementary Figure 4. Height growth potential vs. provenance mean summer precipitation from provenance trials in western North American tree species (Part I: continued in Supp. Fig. 5). Each box represents an independent provenance trial, and each point represents a single population. Slopes are depicted for statistically significant regressions ($p < 0.05$). Species abbreviations consist of the first four letters of the genus, plus the last two letters of the specific epithet (see Fig. 4 for full names). Scale is not comparable among boxes; axes were rescaled independently for maximum visibility of the spread of data.

Supplementary Figure 5. Height growth potential vs. provenance mean summer precipitation from provenance trials in western North American tree species (Part II: continued from Supp. Fig. 4). Each box represents an independent provenance trial,

and each point represents a single population. Slopes are depicted for statistically significant regressions ($p < 0.05$). Species abbreviations consist of the first four letters of the genus, plus the last two letters of the specific epithet (see Fig. 4 for full names). Scale is not comparable among boxes; axes were rescaled independently for maximum visibility of the spread of data.

Supplementary Figure 6. Date of spring events (see text) vs. provenance mean annual temperature from provenance trials in western North American tree species. Each box represents an independent provenance trial, and each point represents a single population. Slopes are depicted for statistically significant regressions ($p < 0.05$). Species abbreviations consist of the first four letters of the genus, plus the last two letters of the specific epithet (see Fig. 4 for full names). Scale is not comparable among boxes; axes were rescaled independently for maximum visibility of the spread of data.

Supplementary Figure 7. Date of spring events (see text) vs. provenance mean summer precipitation from provenance trials in western North American tree species. Each box represents an independent provenance trial, and each point represents a single population. Slopes are depicted for statistically significant regressions ($p < 0.05$). Species abbreviations consist of the first four letters of the genus, plus the last two letters of the specific epithet (see Fig. 4 for full names). Scale is not comparable among boxes; axes were rescaled independently for maximum visibility of the spread of data.

Supplementary Figure 8. Date of fall events (see text) vs. provenance mean annual temperature from provenance trials in western North American tree species. Each box represents an independent provenance trial, and each point represents a single population. Slopes are depicted for statistically significant regressions ($p < 0.05$). Species abbreviations consist of the first four letters of the genus, plus the last two letters of the specific epithet (see Fig. 4 for full names). Scale is not comparable among boxes; axes were rescaled independently for maximum visibility of the spread of data.

Supplementary Figure 9. Date of fall events (see text) vs. provenance mean summer precipitation from provenance trials in western North American tree species. Each box represents an independent provenance trial, and each point represents a single population. Slopes are depicted for statistically significant regressions ($p < 0.05$). Species abbreviations consist of the first four letters of the genus, plus the last two letters of the specific epithet (see Fig. 4 for full names). Scale is not comparable among boxes; axes were rescaled independently for maximum visibility of the spread of data.

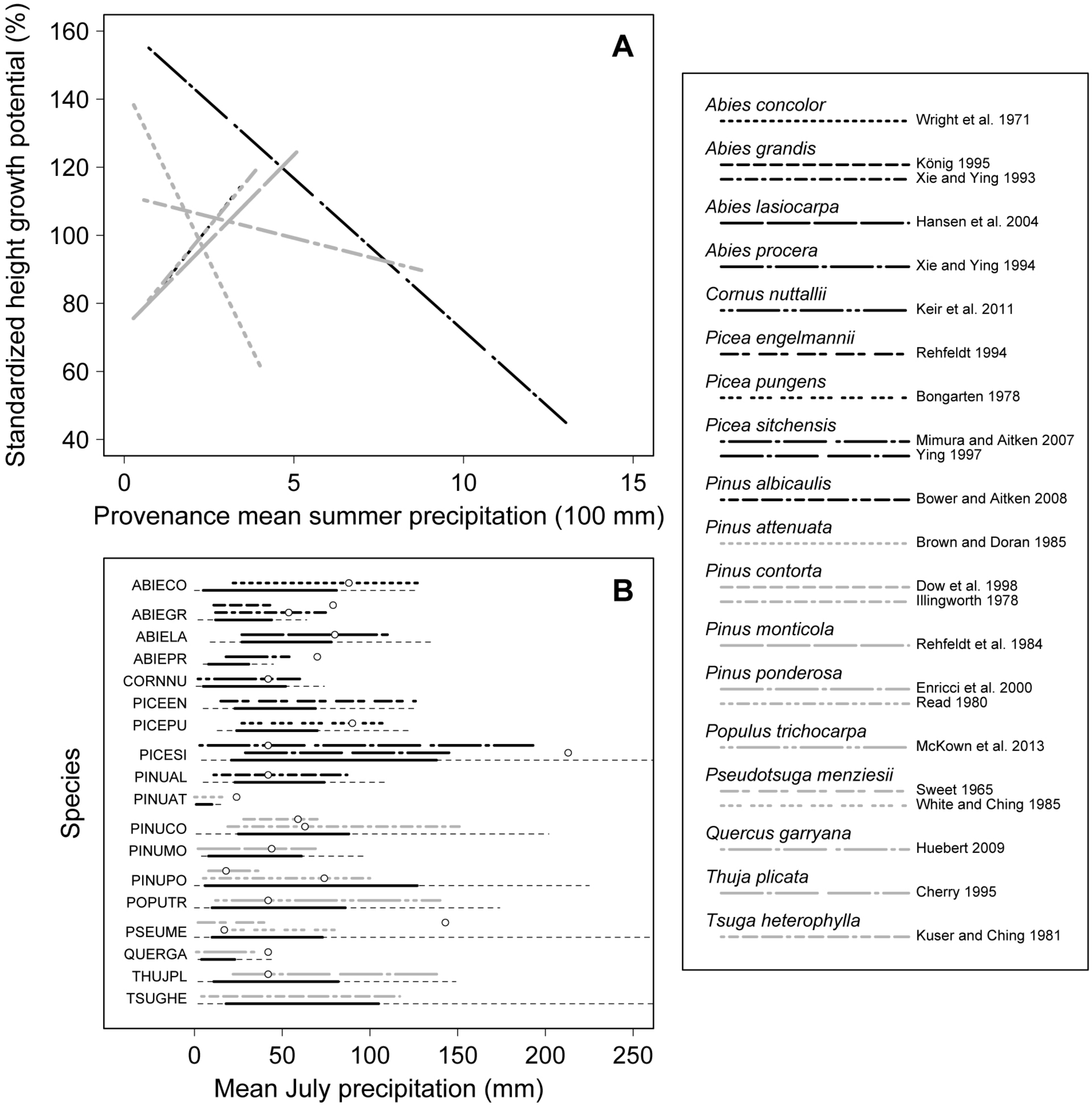

Supplementary Figure 1

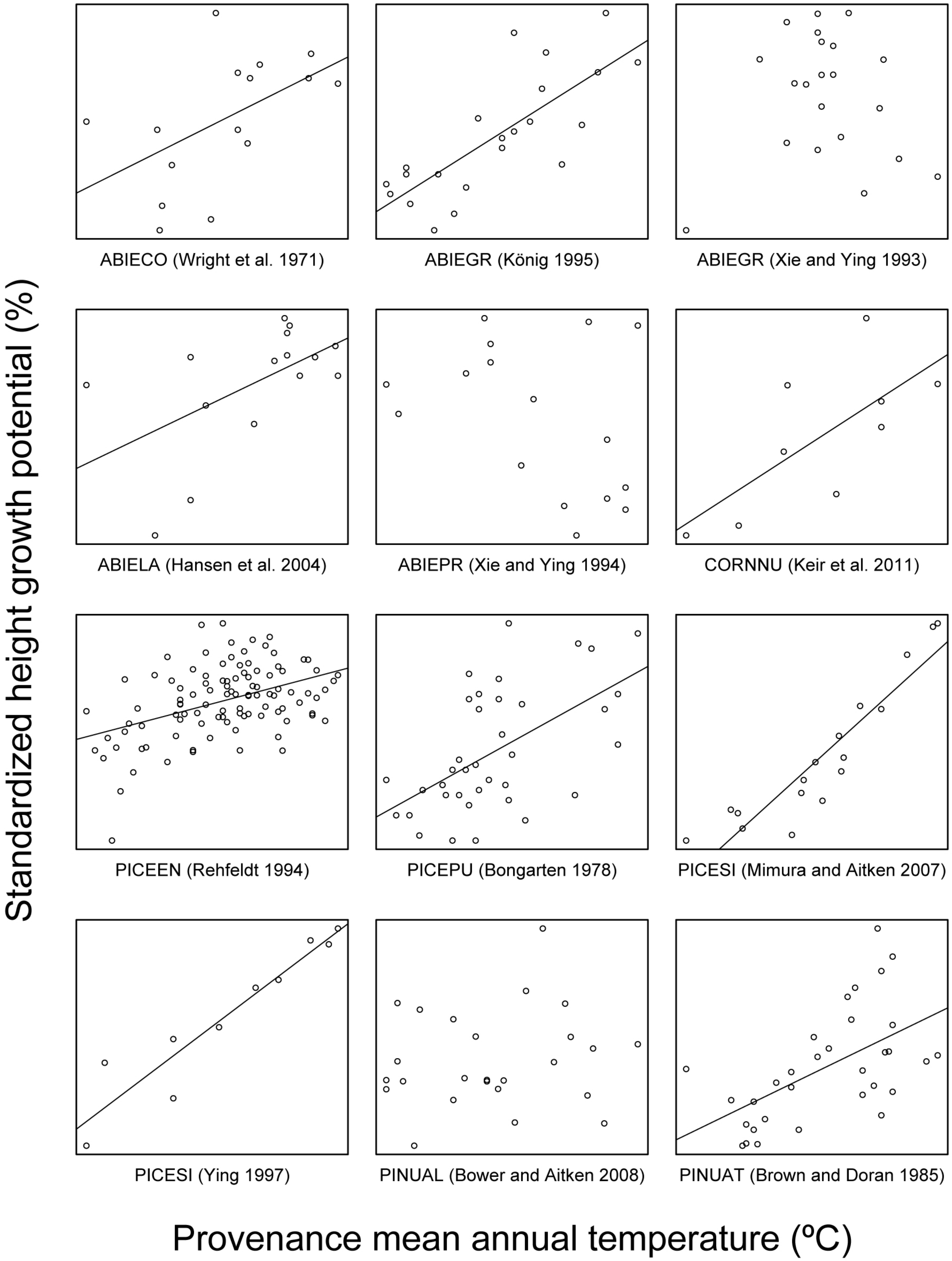

Supplementary Figure 2

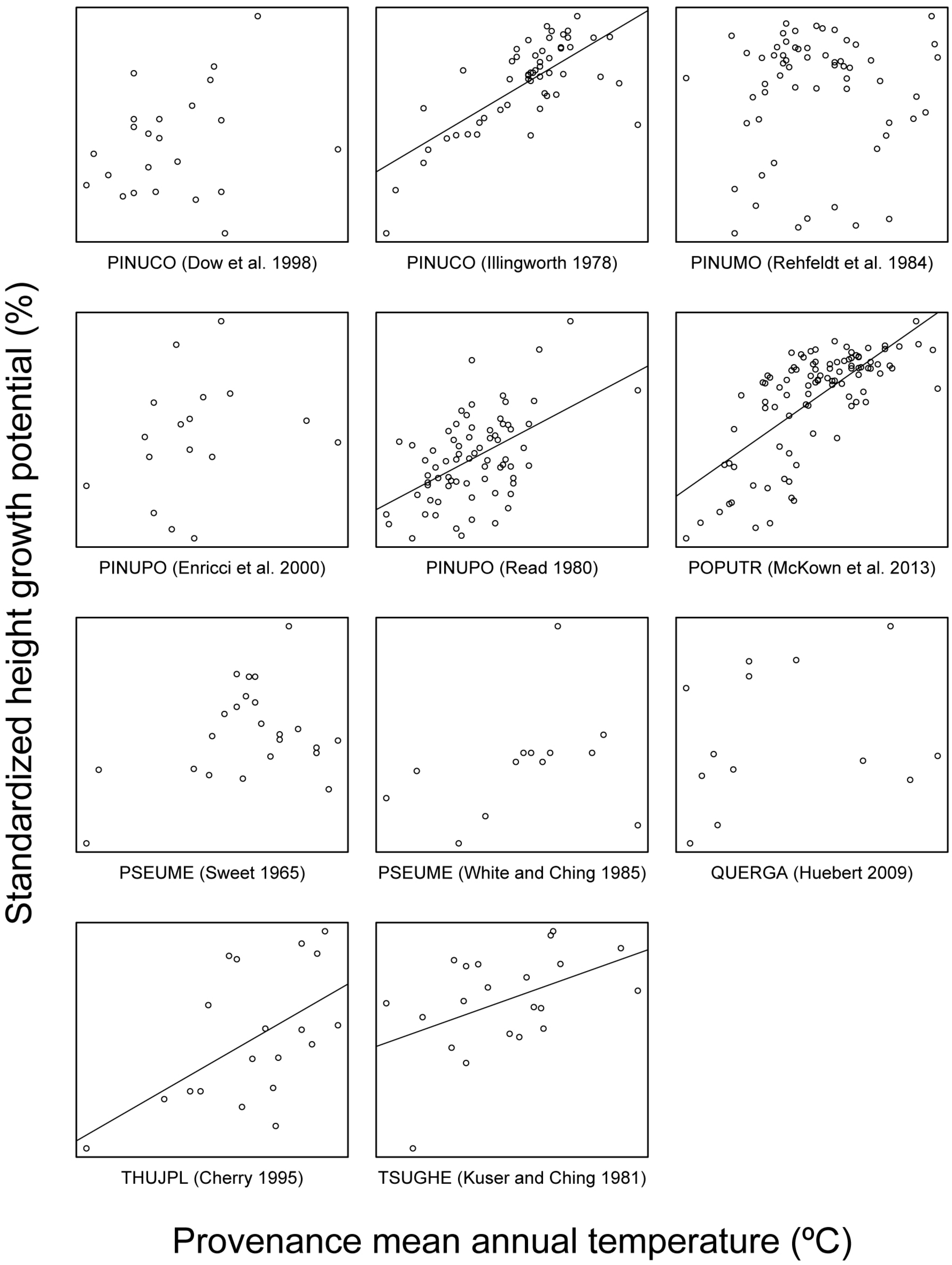

Supplementary Figure 3

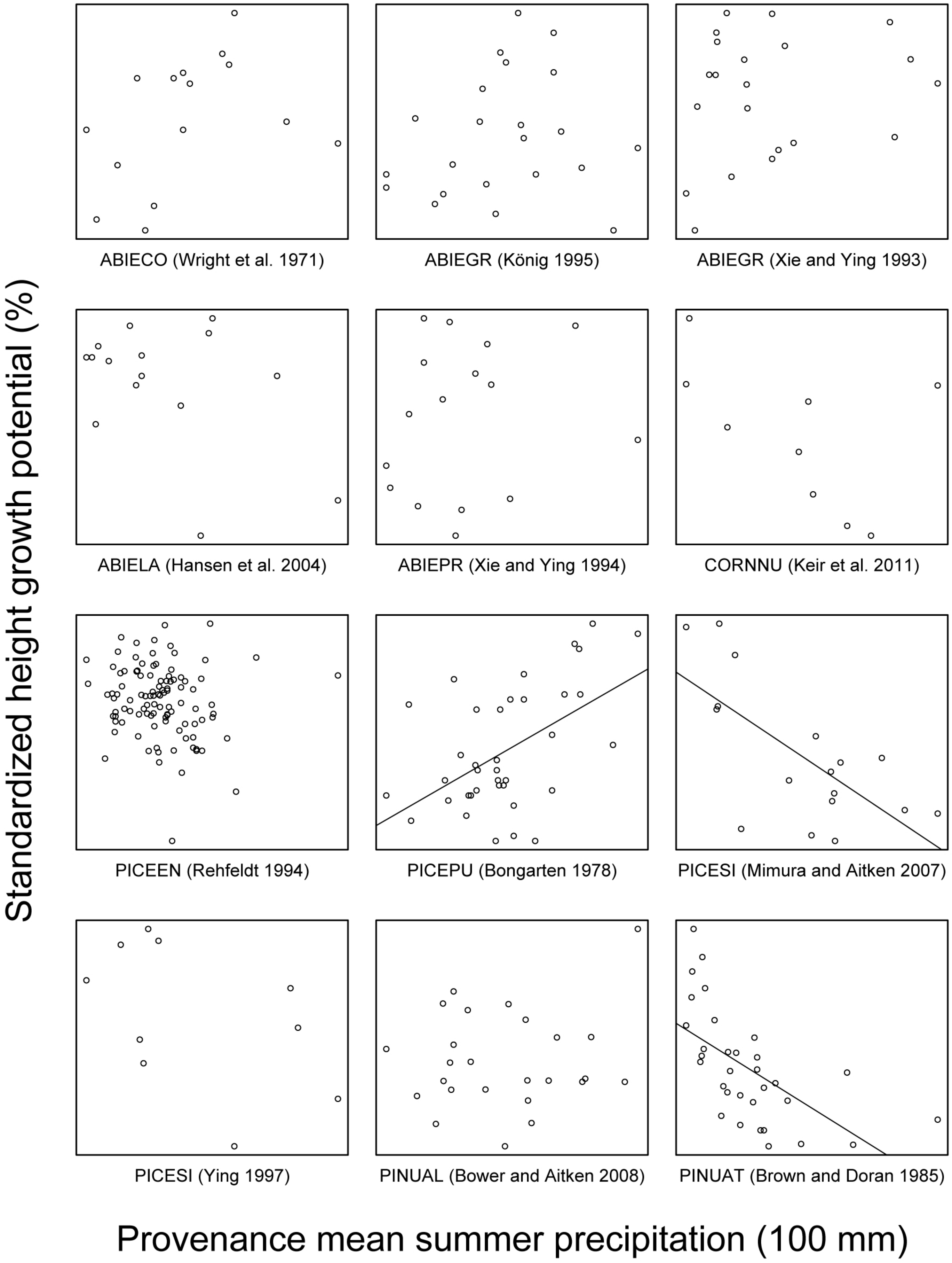

Supplementary Figure 4

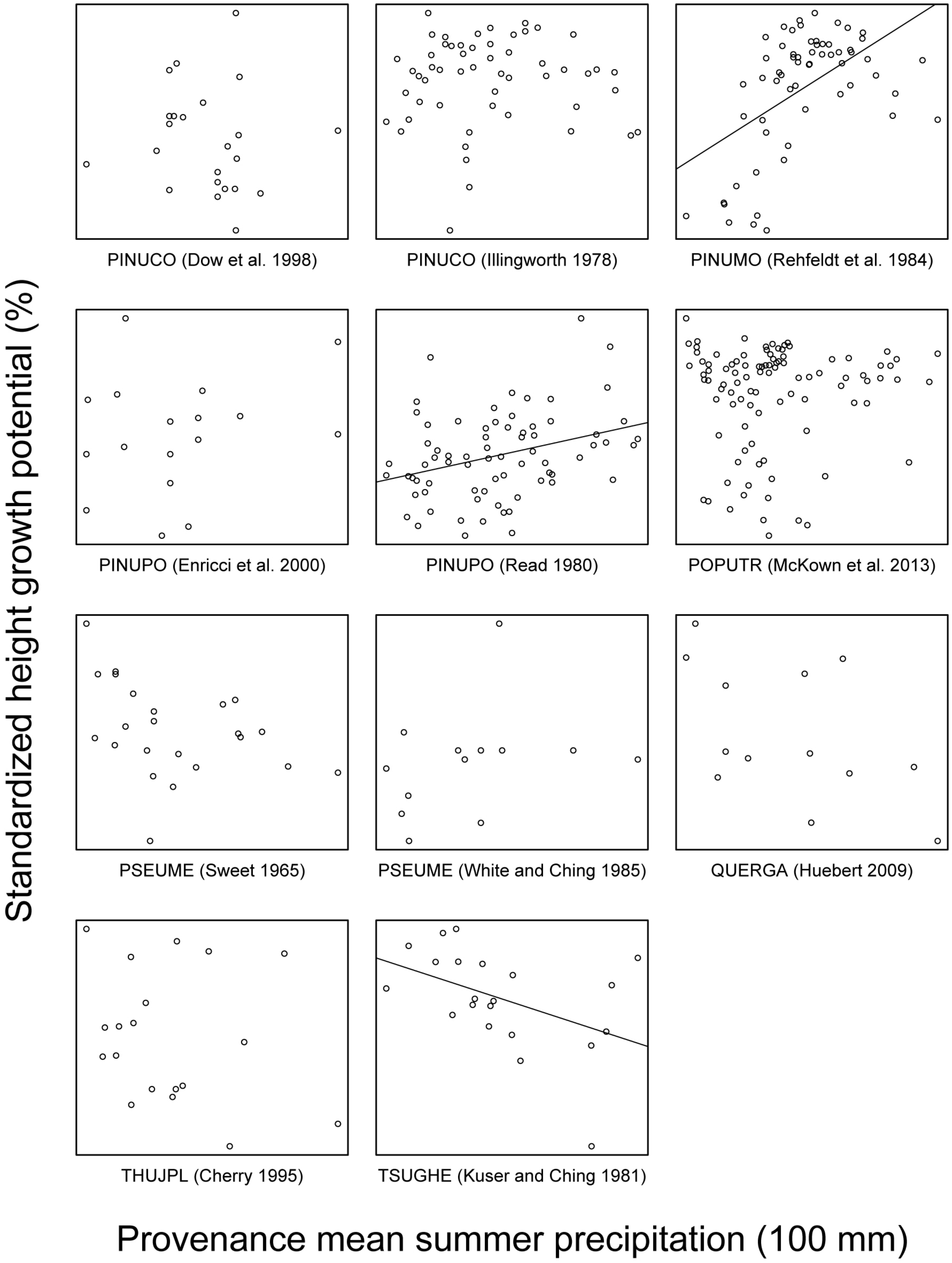

Supplementary Figure 5

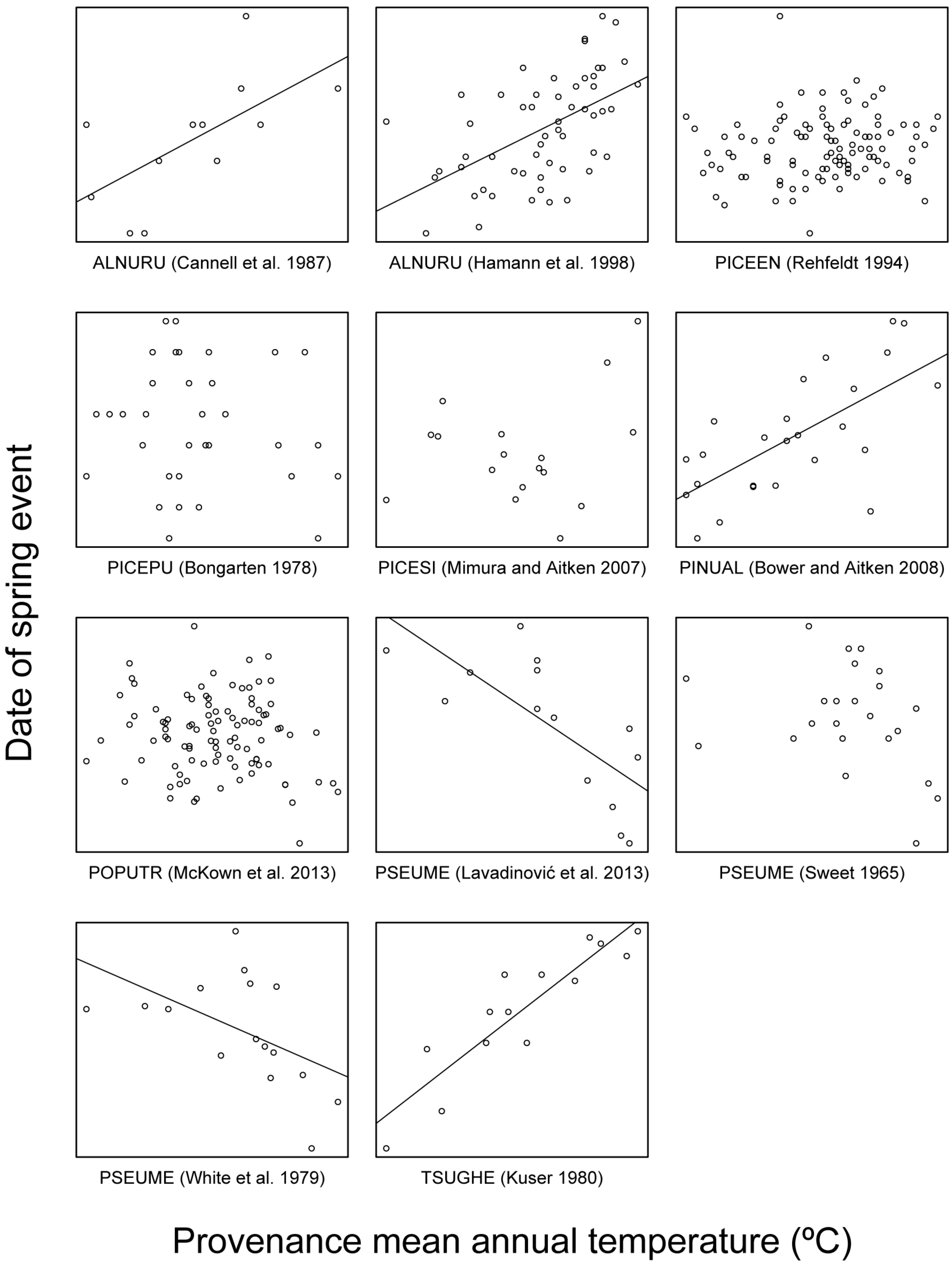

Supplementary Figure 6

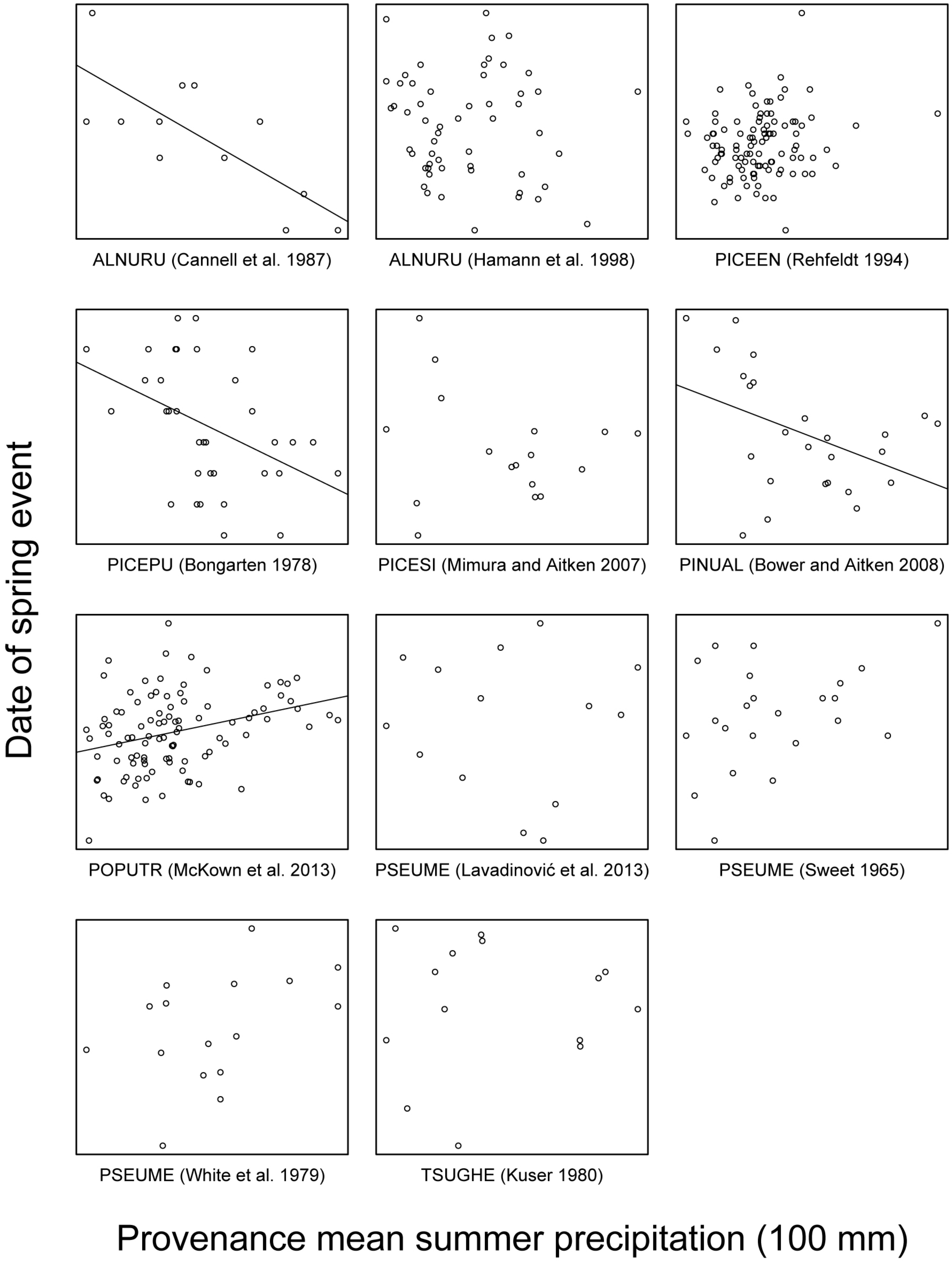

Supplementary Figure 7

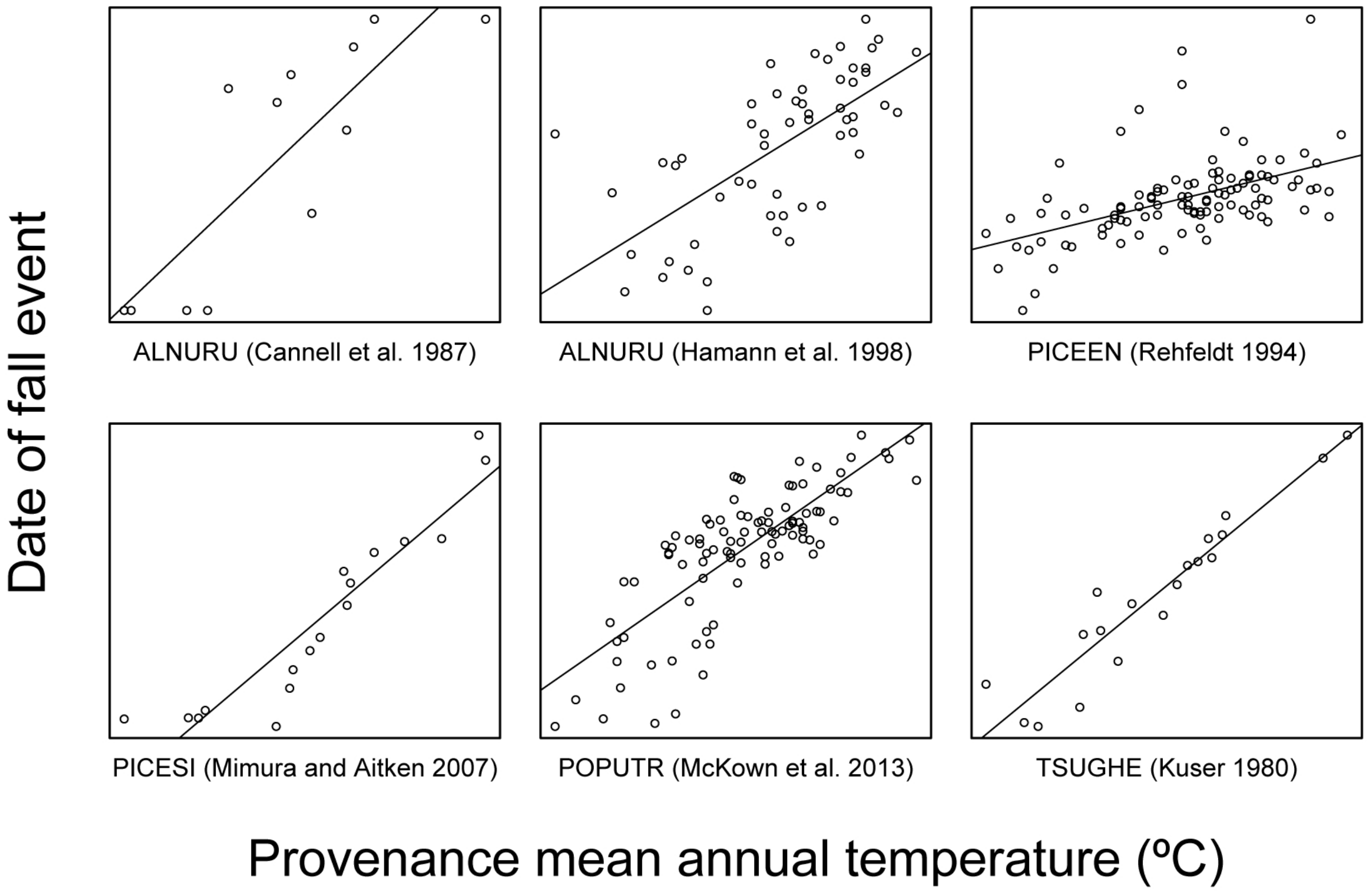

Supplementary Figure 8

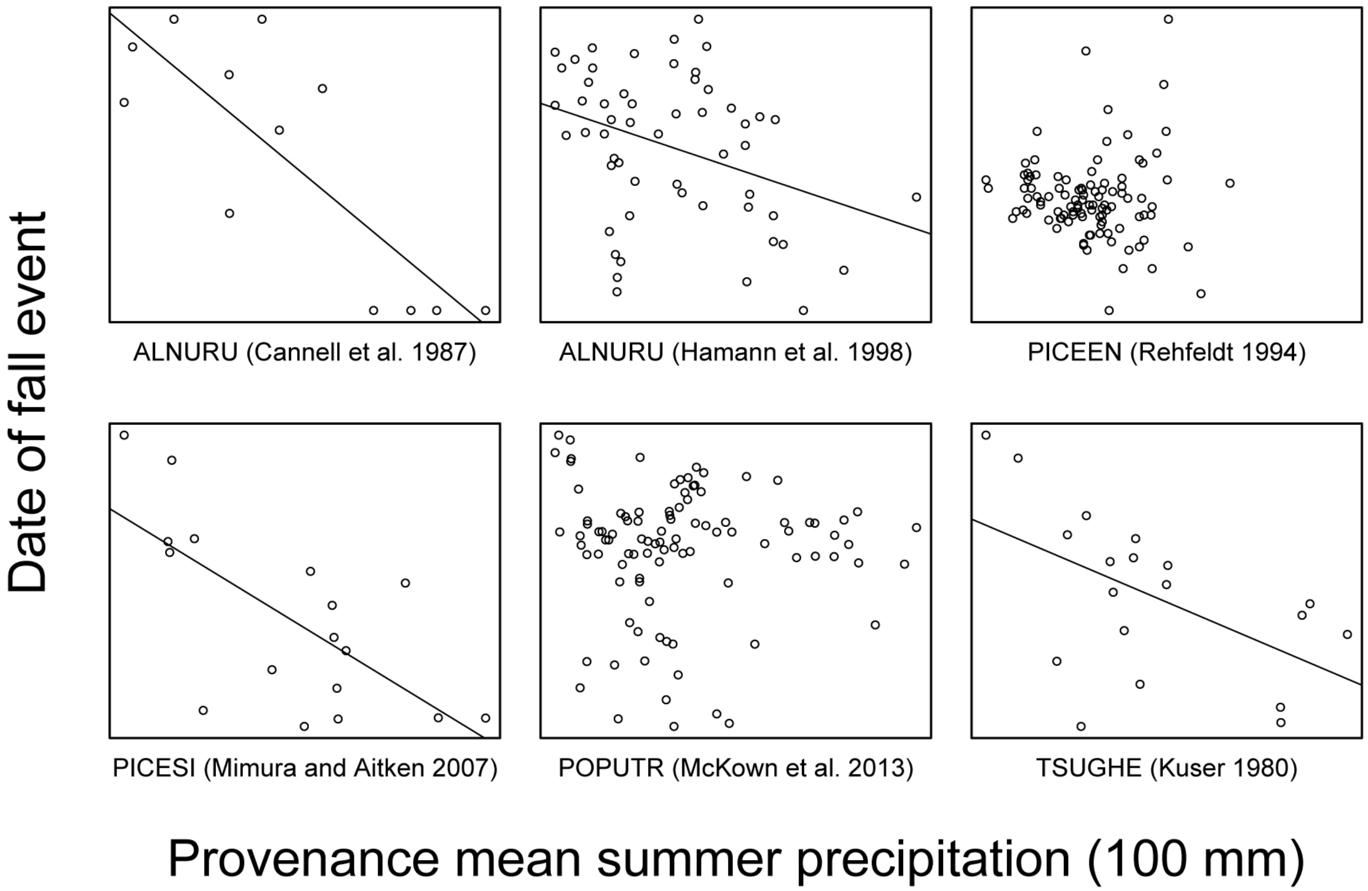

Supplementary Figure 9
